# Diagnostic and differential diagnostic value of plasma extracellular vesicle-associated miRNAs in AIDS patients with tuberculosis and non-tuberculous mycobacterial infection

**DOI:** 10.3389/fcimb.2026.1828046

**Published:** 2026-07-28

**Authors:** Shuibao Xu, Renfang Zhang, Li Liu, Jun Chen, Zhenyan Wang, Tangkai Qi, Jianjun Sun, Wei Song, Yang Tang, Junyang Yang, Youming Chen, Yueming Shao, Jingna Xun, Xinyu Zhang, Zichen Song, Yinzhong Shen, Hongzhou Lu

**Affiliations:** 1 Department of Infectious and Immune Diseases, Shanghai Public Health Clinical Center, Fudan University, Shanghai, China; 2 Department of Infectious Diseases, Shenzhen Third People’s Hospital, Guangdong, China

**Keywords:** aids, extracellular vesicle, HIV, non-tuberculous mycobacteria, tuberculosis

## Abstract

This study evaluates the potential of plasma extracellular vesicle (EV)-associated miRNAs as diagnostic and differential-diagnostic biomarkers to distinguish tuberculosis (TB) from non-tuberculous mycobacterial infection (NTM) among AIDS patients. A cohort of 125 AIDS patients admitted to the Infectious Diseases and Immunology Department of Shanghai Public Health Clinical Center from January 2021 to January 2024 was categorized into AIDS/TB, AIDS/NTM, and AIDS control groups; in the discovery phase, 15 cases (AIDS/TB: 3, AIDS/NTM: 9, AIDS controls: 3) were used to isolate plasma-derived EVs and perform high-throughput sequencing to construct miRNA expression profiles, followed by validation in a large-sample cohort of 110 cases using qPCR. Diagnostic and differential diagnostic value were evaluated with ROC curves, area under the curve (AUC), and the Youden index. In the discovery phase, differentially expressed EV-associated miRNAs were observed among the three groups and among different NTM species; miR-374a-5p and miR-652-3p were markedly downregulated in AIDS/NTM versus AIDS/TB and AIDS controls, while let-7c-5p was upregulated in both AIDS/NTM and AIDS/TB versus AIDS controls. In the validation phase (n=110), miR-374a-5p showed no significant difference between AIDS/TB and AIDS controls but was lower in AIDS/NTM; miR-652-3p was higher in AIDS/TB than controls and lower in AIDS/NTM than controls and AIDS/TB; let-7c-5p was significantly higher in both AIDS/TB and AIDS/NTM than controls, with AIDS/NTM higher than AIDS/TB. ROC analyses yielded: AIDS control vs AIDS/NTM—miR-374a-5p AUC 0.7263, miR-652-3p AUC 0.9947, let-7c-5p AUC 1.0000; AIDS control vs AIDS/TB—miR-374a-5p AUC 0.6800, miR-652-3p AUC 0.7900, let-7c-5p AUC 1.0000; AIDS/NTM vs AIDS/TB—miR-374a-5p AUC 0.8220, miR-652-3p AUC 0.9993, let-7c-5p AUC 0.6613. Collectively, plasma EV-associated miR-374a-5p, miR-652-3p, and let-7c-5p show potential as diagnostic and differential-diagnostic biomarkers for distinguishing TB from NTM infections in AIDS patients, offering a prospective direction for TB/NTM diagnostic strategies in this population.

## 1 Introduction

The genus Mycobacterium comprises Mycobacterium tuberculosis (MTB), NTM, and Mycobacterium leprae. TB and NTM disease are common opportunistic infections in people living with AIDS and are major causes of hospitalization and mortality in this population (AIDS Group, Society of Tropical Diseases and Parasitology, Chinese Medical Association, 2019; Shen et al., 2022).Globally, the infection rate of NTM in AIDS patients is markedly higher than in HIV-negative individuals, with *Mycobacterium avium* being the most prevalent species, followed by *M. intracellulare* and *M. kansasii* (AIDS Group, Society of Tropical Diseases and Parasitology, Chinese Medical Association, 2019; Chinese Society of Tuberculosis, Chinese Medical Association, 2020). In China, similar trends have been reported (AIDS Group, Society of Tropical Diseases and Parasitology, Chinese Medical Association, 2019).In AIDS patients, both TB and NTM disease often present with extrapulmonary involvement, lower rates of cavitary lung lesions, higher rates of disseminated disease, and more frequent non-specific symptoms such as fever, weight loss, and night sweats without localizing signs, compared to HIV-negative individuals (AIDS Group, Society of Tropical Diseases and Parasitology, Chinese Medical Association, 2019; Shen et al., 2022). These two conditions share many clinical features, making differentiation based on clinical presentation alone difficult. Because treatment regimens differ, early diagnosis and treatment are beneficial for prognosis. Currently used methods to distinguish MTB from NTM include p-nitrobenzoic acid medium culture (sensitivity ~30–60% for smear-negative TB, turnaround time 2–6 weeks), MPB64 antigen detection (sensitivity ~70–90%, but requires culture positivity), and PCR-based nucleic acid amplification assays (sensitivity >90% but variable in paucibacillary specimens, costly, requires specialized equipment) (AIDS Group, Society of Tropical Diseases and Parasitology, Chinese Medical Association, 2019; Chinese Society of Tuberculosis, Chinese Medical Association, 2020; Yi et al., 2020). These methods are time-consuming, dependent on lesion-derived specimens, and often not feasible in primary care settings. Therefore, novel non-invasive biomarkers that can be detected in blood are urgently needed. Host miRNAs encapsulated in extracellular vesicles (EVs) are stable in plasma, reflect pathogen-induced immune responses, and can be rapidly quantified by qPCR, offering a potential complementary screening tool for early diagnosis and differentiation of TB and NTM in AIDS patients.

Extracellular vesicles (EVs), including EVs, are nanosized membrane-bound vesicles (40–160 nm) secreted by cells, carrying nucleic acids, proteins, and lipids (Pegtel and Gould, 2019; Jeppesen et al., 2023; Krylova and Feng, 2023; Sonbhadra et al., 2023; Cunha et al., 2024; Hushmandi et al., 2024).They are present in almost all body fluids (plasma, bronchoalveolar lavage fluid, urine) and protect their cargo from degradation (Pegtel and Gould, 2019).A growing body of evidence indicates that EV-associated miRNAs play critical roles in intercellular communication and can modulate host immune responses during infection (Sundar et al., 2019; Wang et al., 2023; Al-Madhagi, 2024; Chen et al., 2024; Mukhtar et al., 2024). We chose to analyze EV-enriched fractions because EV-associated miRNAs are more stable and clinically accessible than free circulating miRNAs. In tuberculosis research, EV-associated miRNAs have shown diagnostic potential (Yu et al., 2016; Smith et al., 2017; Mehaffy et al., 2020; Yan et al., 2021; Ma and Jiu, 2023; Wang et al., 2023; Mukhtar et al., 2024). However, studies on EV-associated miRNAs in NTM infections are limited, especially in the context of HIV co-infection. This study employs high-throughput sequencing to profile plasma EV-associated miRNA expression in AIDS patients with TB or NTM, and validates candidate miRNAs by qPCR as potential non-invasive biomarkers.

The diagnostic significance of EV-associated miRNA in TB is attracting increasing attention. Nevertheless, studies on EVs in relation to NTM are limited.*In vitro* infection studies of macrophages with MTB and NTMs followed by EV proteomic analysis indicate that the protein cargo of macrophage-derived EVs differs after infection with different Mycobacterium species, which may aid in distinguishing species and strains (Zong, 2019). Overall, EVs are widely present and relatively stable in body fluids, are readily enriched and isolated, and hold promise as biomarkers for diagnosing mycobacterial infections.

Nevertheless, most prior studies have focused on HIV-uninfected populations, and there is a paucity of data on changes in EV-associated cargo in AIDS patients following mycobacterial infection. This study employs high-throughput sequencing to define plasma EV-associated miRNA expression profiles, screens for plasma EV-associated miRNAs that may serve as novel molecular diagnostic biomarkers for AIDS/TB and AIDS/NTM, and validates candidates by qPCR to assess diagnostic value. The innovation lies in using plasma EV-associated miRNA as biomarkers to systematically characterize the expression landscape and clinical significance in the setting of AIDS with mycobacterial co-infection. Because EV-associated miRNAs can be detected via a simple plasma test, this approach offers a rapid diagnostic tool to identify infection promptly and inform clinical decisions, with the potential to improve patient prognosis.

## 2 1Materials and methods

### 2.1 Study population

This investigation was conducted at Shanghai Public Health Clinical Center, Shanghai, China, from January 2021 to January 2024.The study comprises two phases: a discovery phase (n=15) in which plasma samples were collected for high-throughput sequencing to establish EV-associated miRNA expression profiles and to identify differentially expressed plasma EV-associated miRNAs between groups; and a validation phase (n=110) in which plasma samples were collected to quantify selected EV-associated miRNAs by qPCR and to evaluate their diagnostic value.

Inclusion criteria: (1) definite AIDS diagnosis; (2) age ≥ 18 years; (3) pathogen-confirmed, treatment-naive TB or NTM disease; (4) voluntary participation. Exclusion criteria: (1) prior anti-TB or anti-NTM therapy; (2) blood samples not meeting study requirements. The study was approved by the Ethics Committee of Shanghai Public Health Clinical Center (2022-S033-01). The requirement for written informed consent was waived by the ethics committee because all plasma samples were anonymized residual clinical specimens, and the study posed no more than minimal risk to participants.

### 2.2 Specimen collection and processing

5 mL peripheral blood was collected in ethylenediaminetetraacetic acid tubes and processed within 1 hour at room temperature or within 2 hours at 4 °C.Centrifuge at 1,900 × g for 10 minutes at 4 °C; transfer the upper plasma to a new sterile tube.Centrifuge the plasma at 3,000 × g for 15 minutes at 4 °C to remove residual debris; transfer the supernatant to a new tube.Aliquot pale yellow plasma and store at −80 °C. Record sample information on a registration form (name, ID, date).

### 2.3 Plasma extracellular vesicle enrichment

Extracellular vesicles (EVs) were enriched from plasma using the exoEasy Maxi Kit (Qiagen, 76064) per the manufacturer’s protocol. This kit enriches a broad population of EVs, including EVs. Plasma was filtered through a 0.8 μm Sartorius Minisart NML membrane. To 4 mL plasma, add 4 mL Buffer XBP, mix, and load onto the exoEasy spin column. Centrifuge at 500 × g for 1 min at room temperature; if liquid remains, spin at 5,000 × g for 1 min. Wash with 10 mL Buffer XWP by centrifuging at 5,000 × g for 5 min, then transfer the column to a new tube. Elute with 400 μL Buffer XE, incubate 1 min and centrifuge at 500 × g for 5 min to collect eluate. For higher yield, re-apply eluate to the same column and repeat until the desired volume. Store the EV solution at −80 °C.

### 2.4 EV identification

#### 2.4.1 Transmission electron microscopy for EV morphology

TEM observations for EV morphology were performed on a Hitachi HT-7800. The sample was washed with PBS three times (15 min each) and fixed in 2% osmium tetroxide at 4 °C for 2 h, followed by three PBS washes. Graded dehydration was conducted in 50%, 70%, 80%, and 90% ethanol (1 mL each, 15 min) and two 100% ethanol washes (20 min each), then two acetone exchanges (1 mL, 15 min each). Infiltration with embedding resin proceeded with acetone:resin ratios of 2:1, 1:1, 1:2 for 2 h, 2 h and overnight, respectively, followed by two exchanges in pure resin (24 h each). Embedded blocks were polymerized at 65 °C for 48 h, then sections were stained with uranyl acetate and lead citrate for 10 min each, rinsed, and examined by TEM.

#### 2.4.2 NTA for EV size determination

Particle size distribution and concentration were determined by nanoparticle tracking analysis (NTA) using a Malvern NanoSight instrument (Malvern Instruments, UK) with Flex software. The sample chamber was cleaned with 200 μL ddH2O and brushed three times, followed by three rinses with ddH2O. After acquisition parameter optimization, a background baseline (BKG) was established. EV suspension (15 μL) was mixed with PBS (200 μL), loaded, and measurements were performed to obtain size distribution and particle concentration.

#### 2.4.3 WB for EV marker proteins

WB was employed to detect EV markers CD9 and CD81, with Calnexin serving as a negative control. Samples were lysed in RIPA buffer containing a protease inhibitor cocktail, and total protein was resolved by SDS–PAGE and transferred to polyvinylidene fluoride membranes. Membranes were probed with primary antibodies against CD9, CD81, and Calnexin (all from Abcam, Shanghai, China) in TBST, washed for 10 minutes, and incubated at room temperature with horseradish peroxidas conjugated secondary antibody (1:10,000; Boster, Wuhan, China) for 1 hour. Signals were visualized by enhanced chemiluminescence. Full, uncropped Western blot membranes are provided in **Supplementary Figure 1**.

### 2.5 miRNA sequencing

miRNA sequencing on the Illumina platform (Shanghai Ni’er Biotechnology Co., Ltd.) was performed to quantify plasma EV–derived miRNA expression and to generate an miRNA expression profile. First, adapter trimming and quality filtering of reads were performed with fastp (v0.20.0) to obtain high-quality clean reads. Clean reads from all samples were merged, and novel miRNAs were predicted with miRDeep2 (v0.1.3). The clean reads were then aligned to a merged human miRNA reference comprising miRBase v22.1 annotations plus the predicted novel miRNAs using Novoalign (v3.09.04) with at most one mismatch. Mapped read counts were defined as the raw expression levels of each miRNA. Counts were normalized across samples using the DESeq2 package (v1.30.1). Differential expression between two groups was assessed based on fold change and p-values.

### 2.6 EV-associated RNA extraction and quality control

250 μL of EV suspension was mixed with 750 μL TRIzol LS. After homogenization, add 200 μL chloroform, cap, and vortex 15 s; incubate 2–3 min at 15–30 °C, then centrifuge at 12,000 × g for 15 min at 4 °C. Transfer the aqueous phase and precipitate RNA with 375 μL isopropanol for 10 min at 15–30 °C, followed by centrifugation at 12,000 × g for 10 min at 4 °C. Wash the pellet with 0.8 mL 75% ethanol, vortex briefly, and centrifuge at 7,500 × g for 5 min at 4 °C. Remove the ethanol, air-dry for 5–10 min, and dissolve RNA in RNase-free water; store at −70 °C.

Quality control and concentration were assessed by NanoDrop ND-1000. Zero with diethyl pyrocarbonate treated water, pipette 1 μL RNA onto the pedestal, and record concentration and purity (A260/280, A260/230). Clean the pedestal after measurement. Acceptable values: A260/280 ≈ 1.8–2.0 and A260/230 > 2.0.

### 2.7 Plasma EV-associated miRNA qPCR detection

#### 2.7.1 Reverse transcription (cDNA synthesis)

Reverse transcription was performed using SuperScript™ III Reverse Transcriptase (Thermo Fisher Scientific) with stem-loop RT primers specific for each miRNA. The RT primer sequences are shown in **Table 1** (new table, see below). The RT reaction mixture (20 μL total) contained: dNTP (2.5 mM each) 2 μL, 10× RT Buffer 2 μL, RT specific primer (1 μM) 0.5 μL, total EV-associated RNA 200 ng, MMLV Reverse Transcriptase (200 U/μL) 1 μL, RNase inhibitor (40 U/μL) 0.5 μL, and RNase-free water up to 20 μL. The RT reaction was performed on a Gene Amp PCR System 9700 (Applied Biosystems) with the following program: 16 °C for 30 min; 60 cycles of 30 °C for 30 s, 42 °C for 30 s, 50 °C for 1 s; followed by 85 °C for 5 min.

**Table 1:** Primer sequences for miRNA qPCR detection.

miRNA	RT primer sequence (5′→3′)	Forward primer (5′→3′)
hsa-miR-374a-5p	GTCGTATCCAGTGCGTGTCGTGGAGTCGGCAATTGCACTGGATACGACCACTTA	GGGTTATAATACAACCTGA
hsa-miR-652-3p	GTCGTATCCAGTGCGTGTCGTGGAGTCGGCAATTGCACTGGATACGACCACAAC	GGGAATGGCGCCACTAGG
hsa-let-7c-5p	GTCGTATCCAGTGCGTGTCGTGGAGTCGGCAATTGCACTGGATACGACAACCAT	GGGTGAGGTAGTAGGTTGT
U6 (internal control)	–	CTCGCTTCGGCAGCACA (forward), AACGCTTCACGAATTTGCGT (reverse)

The universal reverse primer used for all miRNA qPCR was CAGTGCGTGTCGTGGAGT.

#### 2.7.2 Real-time quantitative PCR

qPCR was performed using qPCR SYBR Green Master Mix (CloudSeq) on a QuantStudio 5 Real-Time PCR System (Thermo Fisher Scientific). Primer sequences are listed in **Table 1** (forward primers were designed using Primer 5.0 and synthesized by Yingjun Biotech Co., Ltd.; the universal reverse primer was provided in the SYBR Green kit). The PCR reaction mixture (8 μL per well) contained: 2× SYBR Green Master Mix 5 μL, forward primer (10 μM) 0.5 μL, universal reverse primer (10 μM) 0.5 μL, and RNase-free water to 8 μL. Then 2 μL of cDNA was added to each well of a 384-well PCR plate. The cycling program was: 95 °C for 10 min; 40 cycles of 95 °C for 10 s, 60 °C for 60 s (fluorescence collected); followed by melting curve analysis: 95 °C for 10 s, 60 °C for 60 s, 95 °C for 15 s (ramp rate 0.05 °C/s). All reactions were performed in triplicate. Non-template controls and no-RT controls were included. Relative expression was calculated using the 2^^−ΔΔCt^ method with U6 snRNA as the endogenous control. The raw Ct values and calculated 2^^−ΔΔCt^ values for all individual samples are provided in **Supplementary File 1**.U6 snRNA was selected as the endogenous control because it showed stable expression across all sample groups (Ct variation <1 cycle). We acknowledge that an endogenous miRNA (e.g., miR-16) may be more specific for EV-associated miRNA normalization; this is noted as a limitation.

### 2.8 Data analysis

Statistical analyses were performed using GraphPad Prism 8.0 (GraphPad Software, San Diego, CA, USA) and SPSS Statistics 20.0 (IBM Corp., Armonk, NY, USA). Continuous data are presented as mean ± standard deviation, and categorical data as counts (percentages). For two-group comparisons, independent-samples t-tests were used; for comparisons among three groups, the nonparametric Kruskal–Wallis test was applied to assess differences among groups. Diagnostic performance was evaluated using receiver operating characteristic (ROC) curves and the area under the curve (AUC). All statistical tests were two-sided, and *P* < 0.05 was considered statistically significant.

## 3 Results

### 3.1 EV identification results

#### 3.1.1 EV morphology

**Figure 1:**
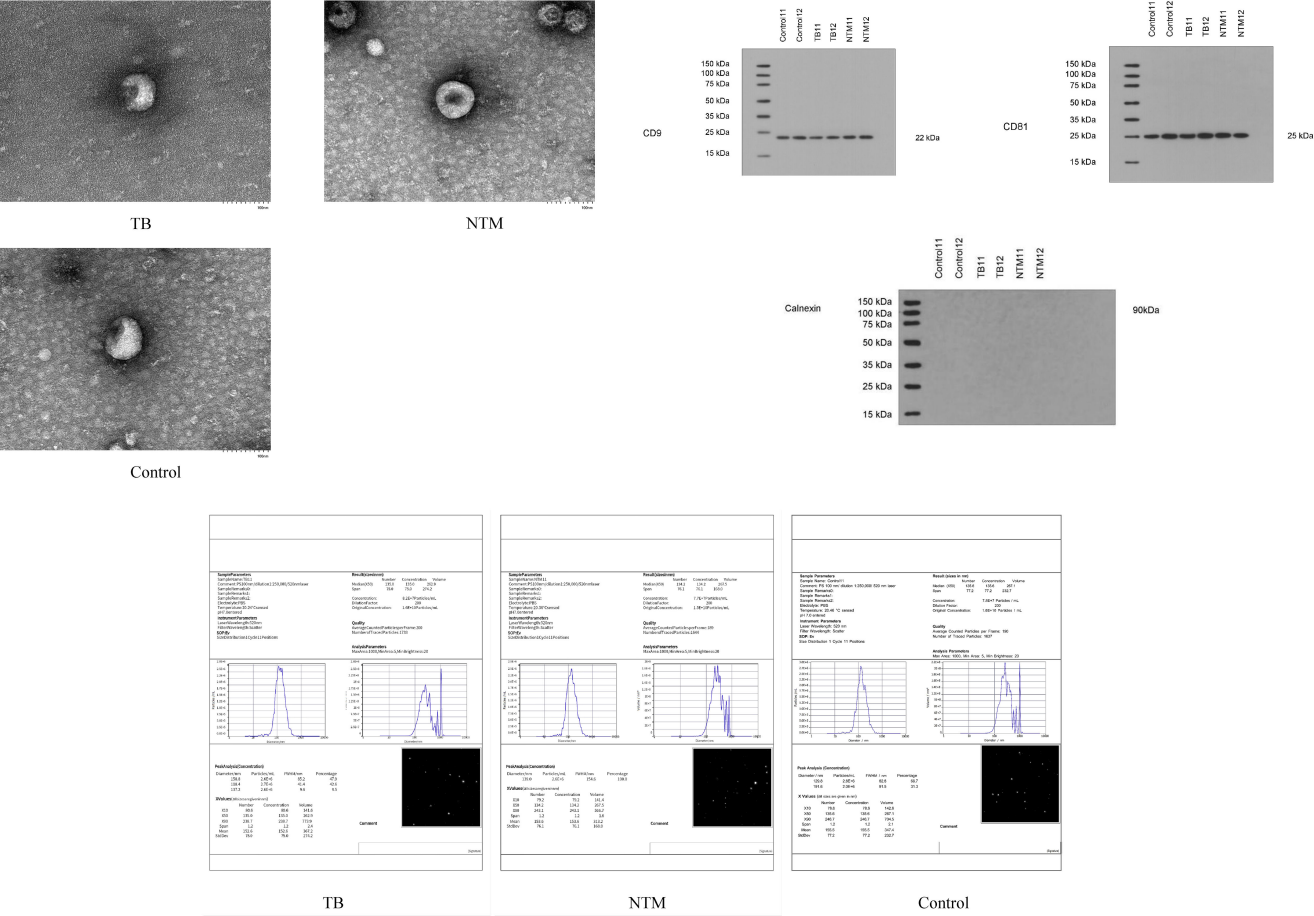
Characterization of plasma EV-enriched fractions. **(A)** Transmission electron microscopy images of EVs from representative AIDS/TB, AIDS/NTM, and AIDS control groups. Scale bar: 100 nm. **(B)** Nanoparticle tracking analysis showing size distribution profiles of EVs from the three groups. X-axis: particle diameter (nm); Y-axis: particle concentration (particles/mL). **(C)** Western blot analysis of EV markers CD9 and CD81; Calnexin was used as a negative control (loading control). Full, uncropped blots are shown in **Supplementary Figure 1**. TB: AIDS/TB, NTM: AIDS/NTM, Control: AIDS control group.

EVs were observed by TEM using a Hitachi HT-7800 and appeared as small vesicles with cup-shaped or biconcave morphologies. TEM images of EVs from the AIDS/TB group, AIDS/NTM group, and AIDS control group are shown in **Figure 1A**, respectively.

#### 3.1.2 EV NTA size distribution

EV concentration and size distribution were measured using a Malvern nanoparticle tracking analyzer. The results are shown in **Figure 1B**. AIDS/TB group EVs had a mean diameter of 152.6 ± 75.0 nm, with three size distribution peaks at 158.8, 118.4 and 137.2 nm, accounting for 47.9%, 42.6% and 9.5% of particles, respectively. AIDS/NTM group EVs had a mean diameter of 153.6 ± 76.1 nm, with a peak at 139.0 nm, representing 100.0% of particles. AIDS control group EVs had a mean diameter of 155.5 ± 77.2 nm, with two peaks at 129.8 and 191.6 nm, representing 68.7% and 31.3% of particles, respectively.

#### 3.1.3 EV WB identification

WB analysis was performed to detect EV-specific protein markers CD9 and CD81.The results are shown in **Figure 1C**.

### 3.2 Plasma EV miRNA sequencing

#### 3.2.1 Baseline clinical characteristics of sequencing patients

miRNA sequencing was performed on samples from 15 patients, including AIDS/TB group (Group A) n=3; AIDS/NTM group n=9, subdivided as Mycobacterium kansasii group (Group B) n=3, Mycobacterium intracellulare group (Group C) n=3, and Mycobacterium avium group (Group D) n=3; AIDS control group (Group E) n=3. Clinical characteristics are shown in **Table 2**. Across the three groups, mean age, CD4+ T cell count, HIV-1 RNA viral load, white blood cell count (WBC), procalcitonin (PCT), C-reactive protein (CRP), and erythrocyte sedimentation rate (ESR) did not differ significantly (*P* > 0.05).

**Table 2:** 15 Basic clinical characteristics of 15 patients undergoing sequencing.

Parameter	AIDS/TB group (n = 3)	AIDS/NTM group (n = 9)	AIDS control group (n = 3)	*P*(K-W test)
Gender (Male %)	66.7%	100%	66.7%	/
Age (Years)	42.00 ± 10.392	42.67 ± 15.612	33.67 ± 12.503	0.505
CD4+ T Lymphocyte Count (cells/μL)	55.33 ± 19.553	27.33 ± 23.054	34.00 ± 9.000	0.213
HIV-RNA *10^4copy/mL	60.13 ± 61.958	29.90 ± 28.736	24.97 ± 26.57	0.567
WBC count*10^9/L	5.74 ± 1.943	4.48 ± 2.86	4.40 ± 1.153	0.685
PCT ng/mL	0.62 ± 1.002	1.81 ± 0.220	0.08 ± 0.065	0.936
CRP mg/L	27.30 ± 17.287	28.69 ± 33.724	12.43 ± 9.550	0.366
ESR mm/h	73.67 ± 29.195	78.44 ± 30.774	53.33 ± 8.327	0.315

WBC, white blood cell count; PCT, procalcitonin; CRP, C-reactive protein; ESR, erythrocyte sedimentation rate; SD, standard deviation; K-W test, Kruskal–Wallis test.

#### 3.2.2 Differential expression screening of EV-associated miRNAs

Plasma-derived EVs were isolated from three groups (AIDS/TB, AIDS/NTM, and AIDS control), comprising a total of 15 patients, and subjected to miRNA sequencing. Pairwise differential expression analysis between groups was performed using the DESeq2 R package. Differentially expressed miRNAs were defined as those with a fold change ≥ 2.0 and a P value ≤ 0.05.

##### 3.2.2.1 Comparison between the AIDS/TB and AIDS/NTM groups

Compared with the combined AIDS/NTM group (Groups B–D), the AIDS/TB group (Group A) exhibited significant upregulation of six plasma EV-derived miRNAs(miR-598-3p, miR-374a-5p, miR-532-3p, miR-652-3p, hsa-miR-576-5p and miR-novel-chr18-15796) and significant downregulation of six others: miR-4732-5p, miR-4732-3p, miR-210-3p, miR-223-3p, miR-125b-5p, and miR-28-3p.

##### 3.2.2.2 Comparison between the AIDS/TB and AIDS control groups

Compared with the AIDS control group (Group E), the AIDS/TB group (Group A) showed significant upregulation of two plasma EV-derived miRNAs (miR-191-5p and let-7c-5p) and significant downregulation of two others: miR-4732-3p and miR-210-3p.

##### 3.2.3.3 Comparison between the AIDS/Kansas mycobacterium and AIDS control groups

Compared with the AIDS control group (Group E), the AIDS/Kansas Mycobacterium group (Group B) exhibited significant upregulation of two plasma EV-derived miRNAs (miR-151a-3p and let-7c-5p) and significant downregulation of 14 others: miR-152-3p, miR-374a-3p, miR-598-3p, miR-novel-chr16-12901, miR-1301-3p, miR-374a-5p, miR-132-3p, miR-889-3p, miR-1273h-3p, miR-652-3p, miR-1306-5p, miR-130b-3p, miR-576-5p, and miR-485-5p.

##### 3.2.3.4 AIDS/intracellular mycobacterium group versus AIDS control group

Compared with the AIDS control group (Group E), the AIDS/intracellular mycobacterium group (Group C) exhibited significant dysregulation of plasma EV–derived miRNAs: six miRNAs were significantly upregulated (miR-191-5p, miR-223-3p, let-7c-5p, miR-126-5p, miR-125b-5p, and miR-206), whereas 87 miRNAs were significantly downregulated, including miR-22-3p, miR-374a-5p, miR-625-3p, and let-7d-3p, among others.

##### 3.2.3.5 AIDS/mycobacterium avium complex group versus AIDS control group

Compared with the AIDS control group (Group E), the AIDS/Mycobacterium avium complex group (Group D) showed significant dysregulation of plasma EV–derived miRNAs: three miRNAs were significantly upregulated (let-7c-5p, miR-483-5p, and miR-125b-5p),whereas 97 miRNAs were significantly downregulated, including miR-493-5p, miR-374a-5p, and miR-652-3p, among others.

##### 3.2.3.6 AIDS/NTM group versus AIDS control group

Based on the aggregated findings from Sections 2.3.3–2.3.5, Mycobacterium avium, Mycobacterium intracellulare, and Mycobacterium kansasii infections were collectively designated as the AIDS/NTM group. Compared with the AIDS control group (Group E), this integrated group exhibited significant dysregulation of plasma EV–derived miRNAs: let-7c-5p was significantly upregulated, whereas miR-374a-5p and miR-652-3p were significantly downregulated.

##### 3.2.3.7 AIDS/mycobacterium kansasii group versus AIDS/mycobacterium intracellulare group

Compared with the AIDS/Mycobacterium intracellulare group (Group C), the AIDS/Mycobacterium kansasii group (Group B) exhibited significant differential expression of plasma EV–derived miRNAs: 63 miRNAs were significantly upregulated—including miR-125a-5p, miR-152-3p, and miR-185-5p—whereas three miRNAs were significantly downregulated: miR-125b-5p, miR-127-3p, and miR-206.

##### 3.2.3.8 AIDS/mycobacterium kansasii group versus AIDS/Mycobacterium avium group

Compared with the AIDS/Mycobacterium avium group (Group D), the AIDS/Mycobacterium kansasii group (Group B) exhibited significant differential expression of plasma EV–derived miRNAs: 44 miRNAs were significantly upregulated, including miR-501-3p, miR-378a-3p, and miR-140-5p, whereas five miRNAs were significantly downregulated: miR-142-5p, hsa-miR-483-5p, hsa-miR-125b-5p, hsa-miR-182-5p, and hsa-miR-574-3p.

##### 3.2.3.9 AIDS/mycobacterium intracellulare group versus AIDS/mycobacterium avium group

Compared with the AIDS/Mycobacterium avium group (Group D), the AIDS/Mycobacterium intracellulare group (Group C) exhibited significant differential expression of plasma EV–derived miRNAs: hsa-miR-127-3p and hsa-miR-206 were significantly upregulated, whereas 24 miRNAs, including miR-125a-5p, miR-185-5p, and miR-142-5p, were significantly downregulated.

Integrated analysis revealed that plasma EV–derived miR-374a-5p and miR-652-3p were significantly downregulated in the AIDS/NTM group—including the Mycobacterium avium, Mycobacterium intracellulare, and Mycobacterium kansasii subgroups—relative to both the AIDS/TB group and the AIDS control group (Group E). In contrast, let-7c-5p was significantly upregulated in both the AIDS/NTM group and the AIDS/TB group compared with the AIDS control group. Collectively, these findings from Phase I identify miR-374a-5p, miR-652-3p, and let-7c-5p as promising diagnostic biomarkers for mycobacterial infection—including both TB and NTM disease—in people living with AIDS. Phase II will validate these candidates using q-PCR to rigorously evaluate their diagnostic sensitivity, specificity, and clinical utility.

### 3.3 qPCR validation results

#### 3.3.1 Baseline clinical characteristics of the qPCR cohort

q-PCR was performed on 110 plasma samples to quantify expression levels of let-7c-5p, miR-374a-5p, and miR-652-3p in EV-enriched fractions. Clinical characteristics of the enrolled participants are summarized in **Table 3**. No statistically significant differences were observed across the three groups in mean age, HIV-1 RNA viral load, PCT, CRP, or ESR. In contrast, CD4+ T lymphocyte count and total WBC count differed significantly among groups (all *P* < 0.05; **Table 3**).

**Table 3:** Baseline clinical characteristics of the qPCR validation cohort (n = 110).

Parameter	AIDS/TB group (n = 30)	AIDS/NTM group (n = 50)	AIDS control group (n = 30)	*P* (K-W test)
Gender (Male %)	100%	94%	96.7%	/
Age (Years)	42.77 ± 12.654	44.43 ± 13.638	38.2 ± 11.412	0.137
CD4+ T Lymphocyte Count (cells/μL)	89.54 ± 71.348	33.39 ± 33.303	104.63 ± 116.908	**0.001**
HIV-RNA *10^4copy/mL	141.45 ± 197.344	94.36 ± 116.371	58.14 ± 51.439	0.201
WBC count*10^9/L	7.50 ± 4.329	4.74 ± 2.704	6.10 ± 2.286	**0.005**
PCT ng/mL	0.30 ± 0.346	5.13 ± 18.649	1.25 ± 2.020	0.083
CRP mg/L	61.57 ± 35.709	49.18 ± 38.669	41.48 ± 29.305	0.061
ESR mm/h	62.39 ± 31.949	65.12 ± 32.354	58.46 ± 28.253	0.549

WBC, white blood cell count; PCT, procalcitonin; CRP, C-reactive protein; ESR, erythrocyte sedimentation rate; SD, standard deviation; K-W test, Kruskal–Wallis test. Bold‑faced P‑values indicate statistically significant differences among three groups with P < 0.05 calculated by Kruskal‑Wallis test.

#### 3.3.2 qRT-PCR quantification of let-7c-5p, miR-374a-5p, and miR-652-3p in plasma EV–enriched fractions

##### 3.3.2.1 Plasma EV–enriched miR-374a-5p

Plasma EV–enriched miR-374a-5p expression did not differ significantly between the AIDS/TB and AIDS control groups (*P* = 0.068). In contrast, expression was significantly downregulated in the AIDS/NTM group relative to both the AIDS control group (*P* < 0.001) and the AIDS/TB group (*P* < 0.001). These findings support the potential of plasma EV–enriched miR-374a-5p as a discriminatory biomarker for NTM infection among people living with AIDS—particularly for differentiating NTM disease from TB.

##### 3.3.2.2 Plasma EV–enriched miR-652-3p

Plasma EV–enriched miR-652-3p expression was significantly upregulated in the AIDS/TB group relative to the AIDS control group (*P* < 0.001), and significantly downregulated in the AIDS/NTM group relative to both the AIDS control group (*P* < 0.001) and the AIDS/TB group (*P* < 0.001). These bidirectional expression patterns—upregulation in TB and downregulation in NTM infection—support the potential of plasma EV–enriched miR-652-3p as a dual-purpose biomarker for both diagnosing mycobacterial infection and discriminating between TB and NTM in people living with AIDS.

##### 3.3.2.3 Plasma EV–enriched let-7c-5p

Plasma EV–enriched let-7c-5p expression was significantly upregulated in both the AIDS/TB group (*P* < 0.001) and the AIDS/NTM group (*P* < 0.001) relative to the AIDS control group; furthermore, expression was significantly higher in the AIDS/NTM group than in the AIDS/TB group (*P* = 0.01). These consistent upregulations—across both mycobacterial infections relative to controls, coupled with a modest but statistically significant elevation in NTM versus TB—suggest that plasma EV–enriched let-7c-5p may serve as a sensitive biomarker for mycobacterial infection in people living with AIDS, while its graded differential expression supports utility in distinguishing NTM from TB.

**Figure 2:**
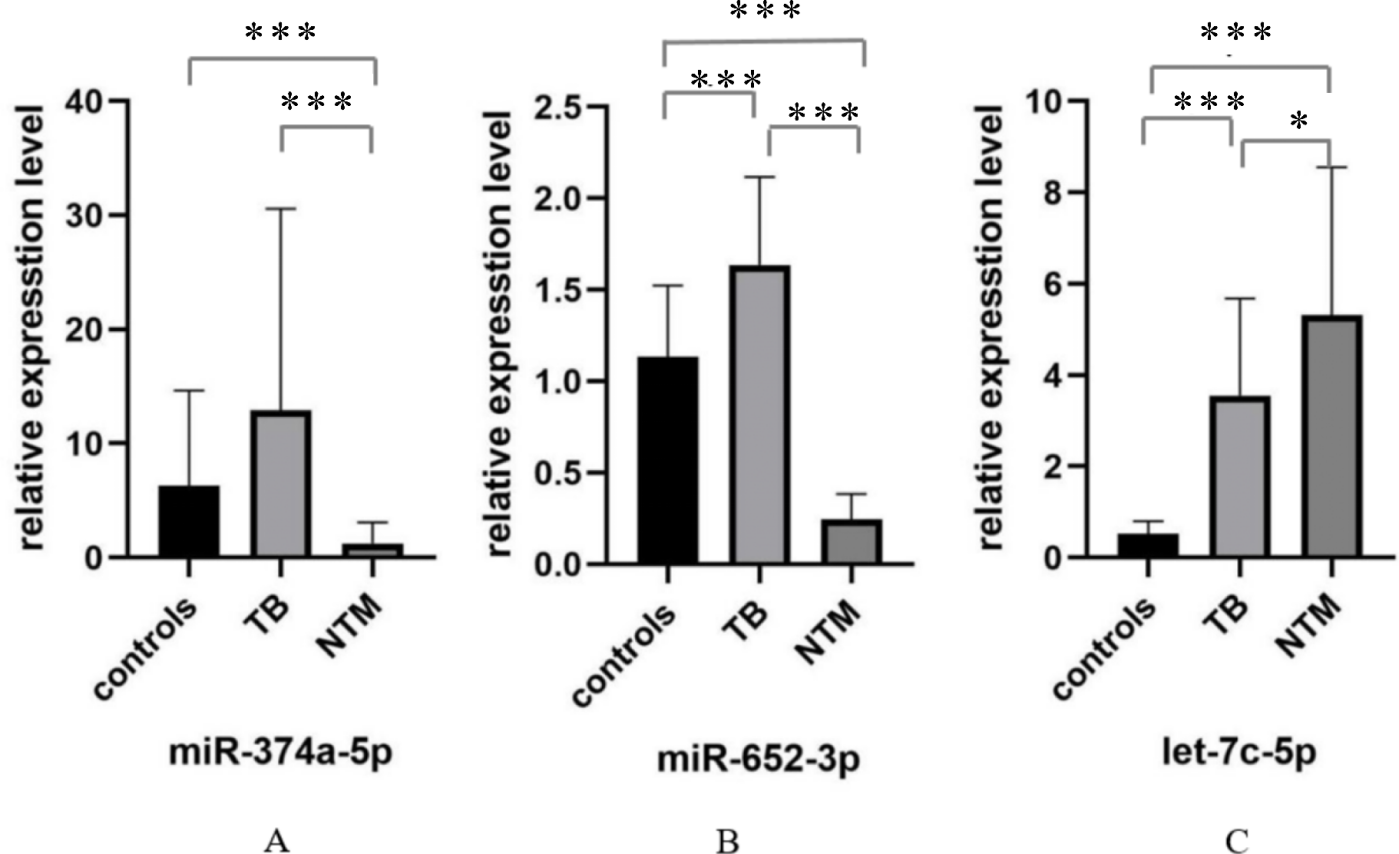
Expression levels of plasma EV-associated miRNAs. qPCR was used to measure the relative expression (2^^−ΔΔCt^), normalized to U6 snRNA and calibrated to the AIDS control group mean) of **(A)** miR-374a-5p, **(B)** miR-652-3p, and **(C)** let-7c-5p in plasma EV-enriched fractions from AIDS control (n=30), AIDS/TB (n=30), and AIDS/NTM (n=50) groups. Horizontal bars indicate the mean ± SD. Statistical significance: *P < 0.05, **P < 0.01, ***P < 0.001 (Kruskal–Wallis test with *post-hoc* pairwise comparisons). Raw Ct values and individual 2^^−ΔΔCt^ values are provided in **Supplementary File 1**.

These findings are summarized in **Table 4** and **Figure 2**. Collectively, plasma EV–enriched let-7c-5p, miR-374a-5p, and miR-652-3p exhibit distinct, infection-specific expression patterns across the AIDS/TB, AIDS/NTM, and AIDS control groups—supporting their potential utility as complementary biomarkers for both detecting mycobacterial infection and differentiating between TB and NTM in people living with AIDS.

**Table 4:** Expression levels of plasma EV miRNAs in AIDS patients with mycobacterial infection.

EV-associated miRNAs	group	n	AVERAGE 2 - △△ CT
miR-374a-5p	AIDS control	30	6.308
	AIDS/TB	30	12.933
	AIDS/NTM	50	1.253
miR-652-3p	AIDS control	30	1.135
	AIDS/TB	30	1.637
	AIDS/NTM	50	0.245
let-7c-5p	AIDS control	30	0.427
	AIDS/TB	30	5.640
	AIDS/NTM	50	6.392

### 3.4 Evaluation of plasma EV-associated miR-374a-5p, miR-652-3p, and let-7c-5p as diagnostic biomarkers for mycobacterial infection in people with AIDS

**Figure 3:**
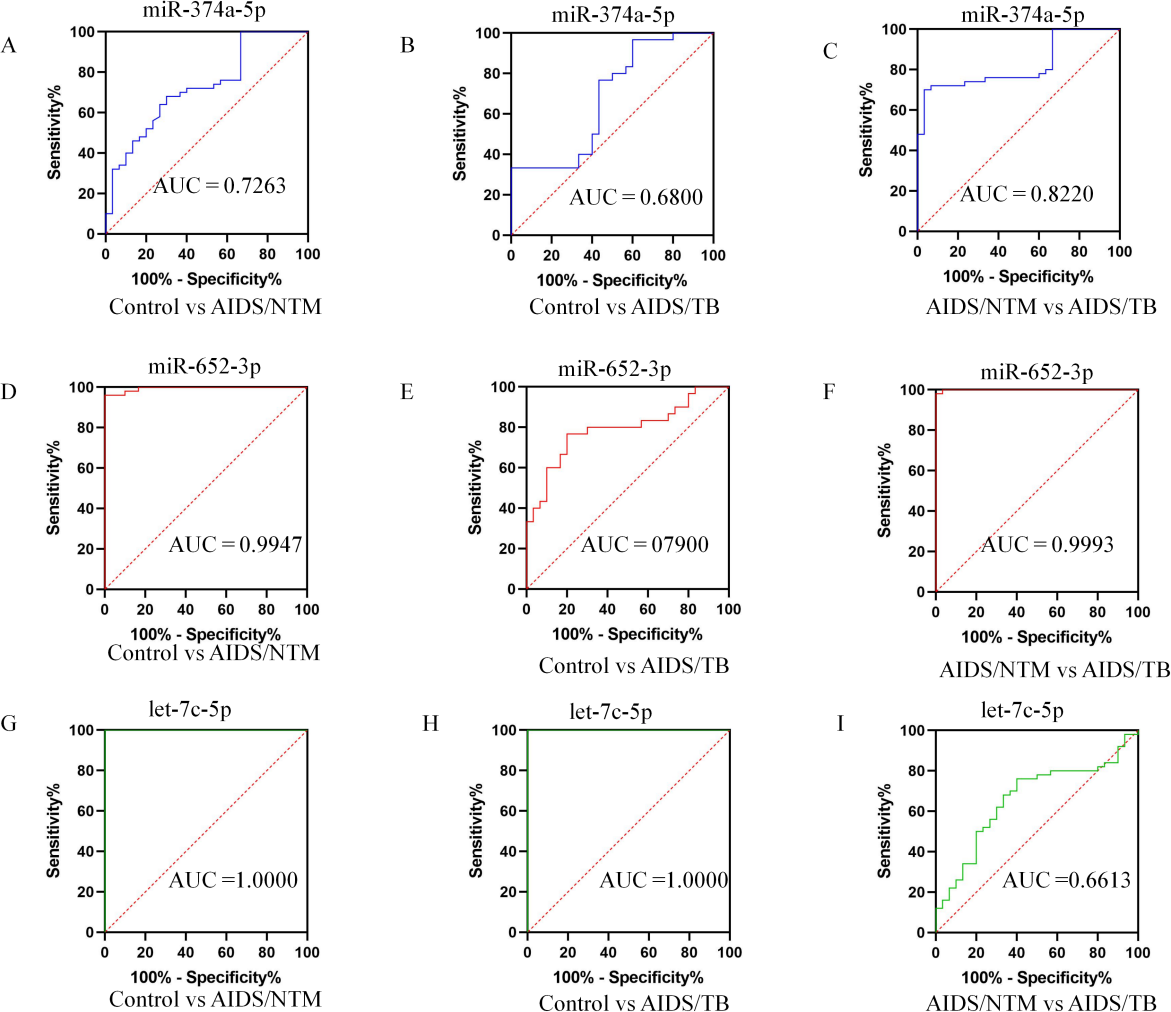
Receiver operating characteristic (ROC) curves of plasma EV-associated miRNAs for discriminating between groups. Each panel shows the ROC curve with area under the curve (AUC) and 95% confidence interval (CI). The diagonal dotted line represents the reference line (AUC = 0.5). **(A)** miR-374a-5p: Control vs AIDS/NTM, AUC = 0.7263 (95% CI 0.6133–0.8394, *P* = 0.0007). **(B)** miR-374a-5p: Control vs AIDS/TB, AUC = 0.6800 (95% CI 0.5426–0.8174, *P* = 0.0166). **(C)** miR-374a-5p: AIDS/NTM vs AIDS/TB, AUC = 0.8220 (95% CI 0.7307–0.9133, *P* < 0.0001). **(D)** miR-652-3p: Control vs AIDS/NTM, AUC = 0.9947 (95% CI 0.9853–1.0000, *P* < 0.0001). **(E)** miR-652-3p: Control vs AIDS/TB, AUC = 0.7900 (95% CI 0.6722–0.9078, *P* = 0.0001). **(F)** miR-652-3p: AIDS/NTM vs AIDS/TB, AUC = 0.9993 (95% CI 0.9971–1.0000, *P* < 0.0001). **(G)** let-7c-5p: Control vs AIDS/NTM, AUC = 1.0000 (95% CI 1.0000–1.0000, *P* < 0.0001). **(H)** let-7c-5p: Control vs AIDS/TB, AUC = 1.0000 (95% CI 1.0000–1.0000, *P* < 0.0001). **(I)** let-7c-5p: AIDS/NTM vs AIDS/TB, AUC = 0.6613 (95% CI 0.5389–0.7837, *P* = 0.0162). Perfect AUC values (1.000) should be interpreted with caution due to limited sample size and potential overfitting; external validation is required.

We constructed ROC curves for plasma EV-associated miR-374a-5p, miR-652-3p, and let-7c-5p to evaluate their diagnostic performance for mycobacterial infection (TB or NTM) in people with AIDS.These findings are summarized in **Table 5** and **Figure 3**.

**Table 5:** ROC results for plasma EV miRNAs across groups.

miRNA	Comparison	AUC	95% CI	cut-off	Youden’s index	*P* value
miR-374a-5p	Control vs AIDS/NTM	0.7263	0.6133–0.8394	0.5090	0.3667	0.0007
miR-374a-5p	Control vs AIDS/TB	0.6800	0.5426–0.8174	0.4595	0.3800	0.0166
miR-374a-5p	AIDS/NTM vs AIDS/TB	0.8220	0.7307–0.9133	0.5040	0.6667	< 0.0001
miR-652-3p	Control vs AIDS/TB	0.7900	0.6722–0.9078	1.296	0.5667	0.0001
miR-652-3p	Control vs AIDS/NTM	0.9947	0.9853–1.0000	0.4530	0.9600	< 0.0001
miR-652-3p	AIDS/NTM vs AIDS/TB	0.9993	0.9971–1.0000	0.4530	0.9800	< 0.0001
let-7c-5p	Control vs AIDS/TB	1.0000	1.0000–1.0000	1.184	1.0000	< 0.0001
let-7c-5p	Control vs AIDS/NTM	1.0000	1.0000–1.0000	1.156	1.0000	< 0.0001
let-7c-5p	AIDS/NTM vs AIDS/TB	0.6613	0.5389–0.7837	3.249	0.3600	0.0162

#### 3.4.1 Comparison between the AIDS control group and the AIDS/NTM group

In this comparison, plasma EV-associated miR-374a-5p yielded an AUC of 0.7263 (95% CI 0.6133–0.8394; *P* = 0.0007; **Figure 3A**), with a cut-off of 0.5090 and the best sensitivity and specificity of 96.67% and 40%, respectively (Youden’s index = 0.3667). Plasma EV-associated miR-652-3p yielded an AUC of 0.9947 (95% CI 0.9853–1.0000; *P* < 0.0001; **Figure 3D**) with a cut-off of 0.4530, sensitivity 96%, specificity 100% (Youden’s index = 0.9600). Plasma EV-associated let-7c-5p yielded an AUC of 1.0000 (95% CI 1.0000–1.0000; *P* < 0.0001; **Figure 3G**) with a cut-off of 1.156, and both sensitivity and specificity 100% (Youden’s index = 1.0000).The AUC for let-7c-5p reached 1.0000 (95% CI 1.0000–1.0000; *P* < 0.0001), indicating perfect discrimination between the AIDS control and AIDS/NTM groups in this cohort. However, given the limited sample size and potential overfitting, external validation in independent cohorts is required.

#### 3.4.2 Comparison between the AIDS control group and the AIDS/TB group

In this comparison, plasma EV-associated miR-374a-5p yielded an AUC of 0.6800 (95% CI 0.5426–0.8174; *P* = 0.0166; **Figure 3B**), with a cut-off of 0.4595 and the best sensitivity and specificity of 68.0% and 70.0%, respectively (Youden’s index = 0.3800); plasma EV-associated miR-652-3p yielded an AUC of 0.7900 (95% CI 0.6722–0.9078; *P* = 0.0001; **Figure 3E**), with a cut-off of 1.296 and sensitivity 76.67% and specificity 80.0% (Youden’s index = 0.5667); plasma EV-associated let-7c-5p yielded an AUC of 1.0000 (95% CI 1.0000–1.0000; *P* < 0.0001; **Figure 3H**), with a cut-off of 1.184 and both sensitivity and specificity 100% (Youden’s index = 1.0000). *Note: as in 4.1, an AUC of 1.0000 should be interpreted with caution due to potential overfitting and limited sample size; external validation in independent cohorts is required.*

#### 3.4.3 Comparison between the AIDS/NTM and the AIDS/TB groups

In this comparison, plasma EV-associated miR-374a-5p yielded an AUC of 0.8220 (95% CI 0.7307–0.9133; *P* < 0.0001; **Figure 3C**), with a cut-off of 0.5040 and the best sensitivity and specificity of 70.0% and 96.7%, respectively (Youden’s index = 0.6667); plasma EV-associated miR-652-3p yielded an AUC of 0.9993 (95% CI 0.9971–1.0000; *P* < 0.0001; **Figure 3F**), with a cut-off of 0.4530 and sensitivity 98.0% and specificity 100.0% (Youden’s index = 0.9800); plasma EV-associated let-7c-5p yielded an AUC of 0.6613 (95% CI 0.5389–0.7837; *P* = 0.0162; **Figure 3I**), with a cut-off of 3.249 and sensitivity 76.0% and specificity 60.0% (Youden’s index = 0.3600).

These findings suggest that plasma EV-associated miR-374a-5p, miR-652-3p, and let-7c-5p are differentially expressed between AIDS patients with TB and those with NTM infection. While not definitive for differential diagnosis, these miRNAs may serve as complementary, non-invasive biomarkers to help distinguish the two conditions.

## 4 Discussion

In this study, we profiled plasma EV-associated miRNA expression in AIDS patients with TB or NTM infection and validated three miRNAs (miR-374a-5p, miR-652-3p, let-7c-5p) as potential complementary biomarkers. Our findings are consistent with several previous reports. The let-7c-5p upregulation in both TB and NTM groups aligns with studies showing let-7 family involvement in mycobacterial infection (Kumar et al., 2015; Zhan et al., 2022). Kumar et al. demonstrated that let-7 modulates the immune response to M. tuberculosis by targeting A20, an inhibitor of NF-κB (Kumar et al., 2015). Similarly, Zhan et al. reported that mmu-let-7c-5p was specifically upregulated in macrophage-derived EVs after BCG infection (Zhan et al., 2022). The upregulation of let-7c-5p in both TB and NTM suggests a common host response to mycobacterial infection, while its higher level in NTM versus TB (P = 0.01) may reflect species-specific immune modulation. However, the AUC for let-7c-5p distinguishing NTM from TB was only 0.6613, indicating limited utility for differential diagnosis alone.

MiR-652-3p showed bidirectional regulation: upregulation in TB and downregulation in NTM relative to controls. To our knowledge, this is the first report of miR-652-3p expression in mycobacterial infections. Stevens and Saunders reviewed that miR-652-3p targets LLGL1, ZEB1, and JAG1, regulating cell polarity, metastasis, and angiogenesis (Stevens and Saunders, 2021). The opposite expression patterns in TB versus NTM suggest that miR-652-3p may be involved in divergent host-pathogen interactions, though the mechanism remains unknown.

MiR-374a-5p was downregulated in NTM but not significantly changed in TB compared to controls. This finding partially agrees with Cui et al., who reported that EV-associated miR-374a-5p is specifically upregulated in latent TB infection (Cui et al., 2024). The discrepancy may be due to differences in disease stage (active vs. latent) or the HIV co-infection status of our cohort. In inflammatory bowel disease, miR-374a-5p suppresses pro-inflammatory genes and monocyte migration (Perez-Sanchez et al., 2022), suggesting a possible anti-inflammatory role that may be differentially modulated by TB versus NTM.

It is important to emphasize that host-derived miRNA signatures cannot replace pathogen-based diagnostic methods. Instead, they may serve as complementary tools to raise clinical suspicion or guide further testing, particularly when lesion specimens are difficult to obtain. The perfect AUC values (1.000) observed for let-7c-5p in control vs. infection comparisons are promising but should be interpreted with caution due to limited sample size and potential overfitting. Complete separation in small-sample analyses may overestimate generalizability.

### 4.1 Limitations

This study has several limitations. (1) The sample size is modest, particularly for subgroup comparisons yielding perfect AUC values, raising the possibility of overfitting; external validation is required. (2) The exoEasy Maxi Kit enriches total EVs rather than pure EVs; thus, our findings reflect EV-associated miRNAs. (3) We did not perform orthogonal methods (e.g., FACS) to quantify EV purity. (4) RNA was extracted using TRIzol LS, which may have lower efficiency for small RNAs than column-based kits. (5) U6 snRNA was used as the normalizer; an endogenous miRNA might be more appropriate. (6) The cohort consisted of HIV-infected individuals, limiting generalizability to HIV-negative populations. (7) Different NTM species were not separately validated due to small subgroup sizes.

### 4.2 Future perspectives

Multi-center, large-scale prospective studies are needed to validate these findings in independent cohorts, including HIV-negative individuals. Future work should also compare extraction methods, evaluate dynamic changes during treatment, and integrate proteomic and functional assays to elucidate mechanisms.

## 5 Conclusion

In conclusion, plasma EV-associated miR-374a-5p, miR-652-3p, and let-7c-5p show differential expression patterns in AIDS patients with TB versus NTM infection. While pathogen-based methods remain the gold standard for definitive differential diagnosis, these host-derived miRNA signatures may serve as complementary, non-invasive biomarkers to aid early suspicion and guide diagnostic workup. Future large-scale, multi-center validation studies are needed to confirm their clinical utility.

## Data Availability

The datasets supporting the findings of this study are available in the Zenodo repository (DOI: https://doi.org/10.5281/zenodo.21161322). Metadata is open to the public, and raw experimental data including anonymized clinical information, miRNA differential expression results, qPCR data and original WB images can be accessed by editors and reviewers through the confidential link in the reviewer response letter. After article acceptance, all data will be released publicly.
