# Supplementary material for: Diagnostic and differential diagnostic value of plasma extracellular vesicle-associated miRNAs in AIDS patients with tuberculosis and non-tuberculous mycobacterial infection
Source: Front Cell Infect Microbiol. 2026 Jul 28;16:1828046. doi: 10.3389/fcimb.2026.1828046 (PMC13456989; doi:10.3389/fcimb.2026.1828046)
Supplement: Supplementary file 1 [file SupplementaryFile1.pdf]

Sample Parameters
Sample Name: Control11
Comment: PS 100 nm/ dilution 1:250,000/ 520 nm laser
Sample Remarks0:
Sample Remarks1:
Sample Remarks2:
Electrolyte: PBS
Temperature: 20.46 °C sensed
pH 7.0 entered

Instrument Parameters
Laser Wavelength: 520 nm
Filter Wavelength: Scatter

SOP: Ev
Size Distribution 1 Cycle 11 Positions

Result (sizes in nm)

	Number	Concentration	Volume
Median (X50)	135.6	135.6	267.1
Span	77.2	77.2	232.7

Concentration: 7.8E+7 Particles / mL
Dilution Factor: 200
Original Concentration: 1.6E+10 Particles / mL

Quality
Average Counted Particles per Frame: 190
Number of Traced Particles: 1637

Analysis Parameters
Max Area: 1000, Min Area: 5, Min Brightness: 20

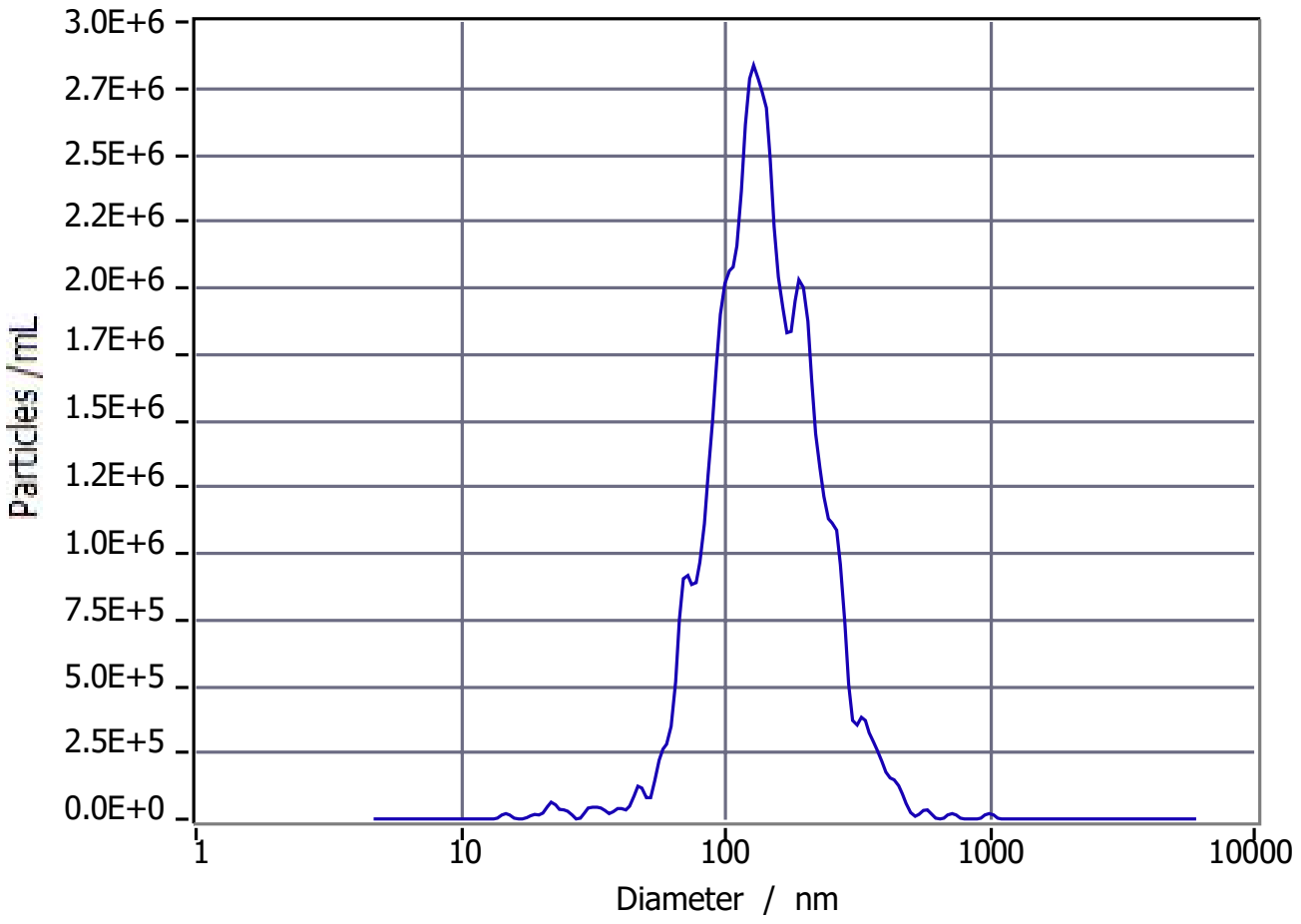

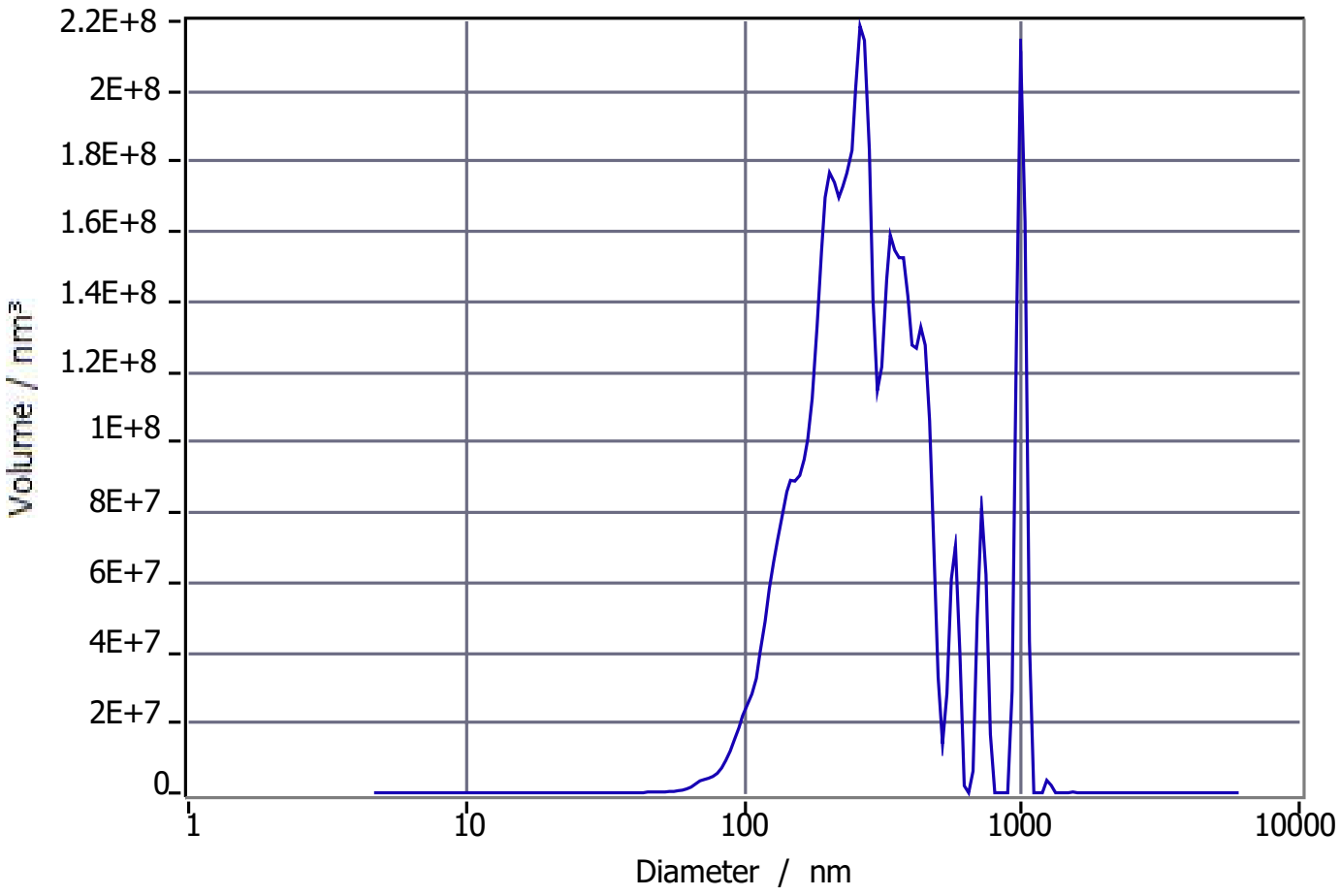

Peak Analysis (Concentration)

Diameter / nm	Particles/mL	FWHM / nm	Percentage
129.8	2.8E+6	82.8	68.7
191.6	2.0E+6	91.5	31.3

X Values (all sizes are given in nm)

	Number	Concentration	Volume
X10	78.6	78.6	142.6
X50	135.6	135.6	267.1
X90	246.7	246.7	704.5
Span	1.2	1.2	2.1
Mean	155.5	155.5	347.4
StdDev	77.2	77.2	232.7

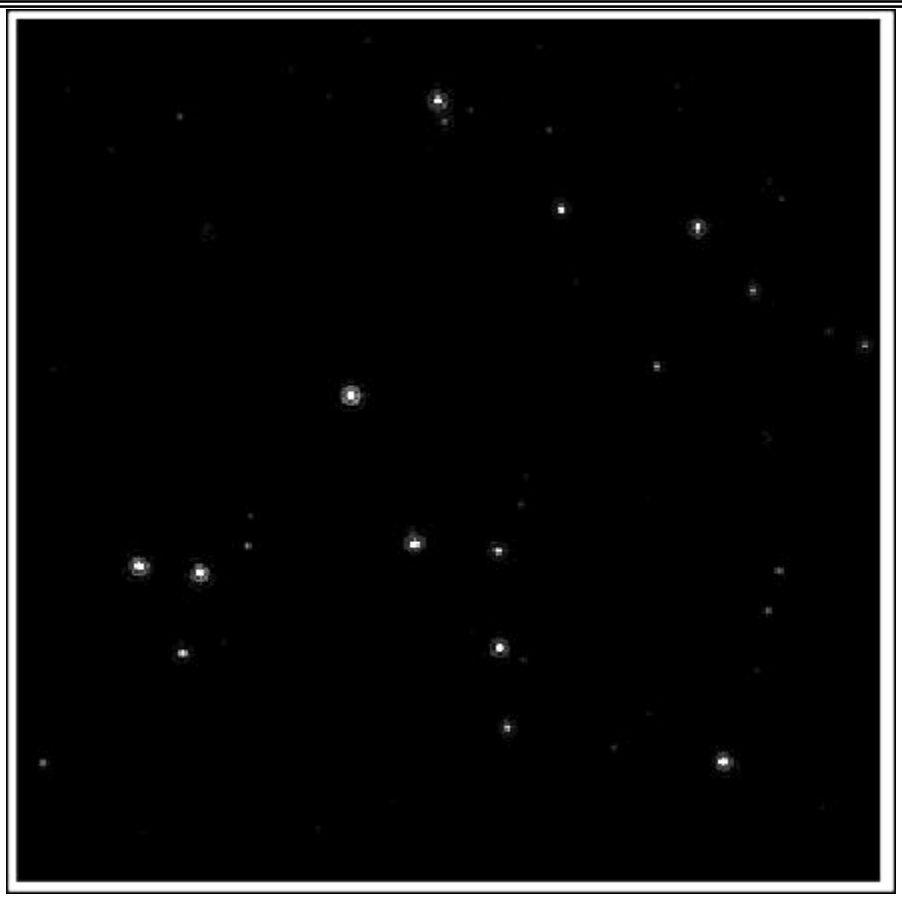

Comment

(Signature)
