# Supplementary material for: Diagnostic and differential diagnostic value of plasma extracellular vesicle-associated miRNAs in AIDS patients with tuberculosis and non-tuberculous mycobacterial infection
Source: Front Cell Infect Microbiol. 2026 Jul 28;16:1828046. doi: 10.3389/fcimb.2026.1828046 (PMC13456989; doi:10.3389/fcimb.2026.1828046)
Supplement: Supplementary file 2 [file SupplementaryFile2.pdf]

SampleParameters
SampleName:NTM11
Comment:PS100nm/dilution1:250,000/520nmlaser
SampleRemarks0:
SampleRemarks1:
SampleRemarks2:
Electrolyte:PBS
Temperature:20.36°Csensed
pH7.0entered

InstrumentParameters
LaserWavelength:520nm
FilterWavelength:Scatter

SOP:Ev
SizeDistribution1Cycle11Positions

Result(sizesinnm)

	Number	Concentration	Volume
Median(X50)	134.2	134.2	267.5
Span	76.1	76.1	168.0

Concentration: 7.7E+7Particles/mL
DilutionFactor: 200
OriginalConcentration: 1.5E+10Particles/mL

Quality
AverageCountedParticlesperFrame:189
NumberofTracedParticles:1644

AnalysisParameters
MaxArea:1000,MinArea:5,MinBrightness:20

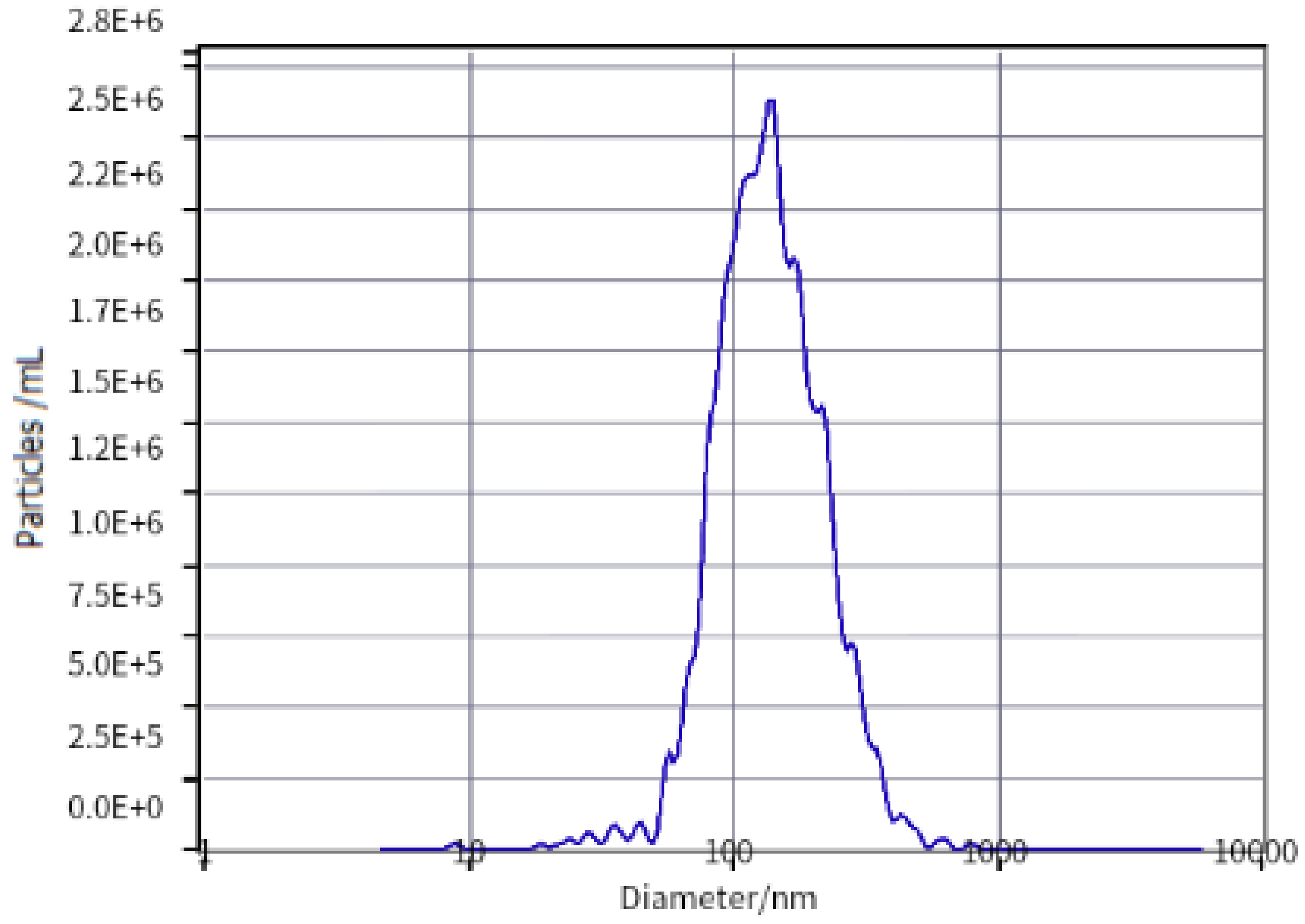

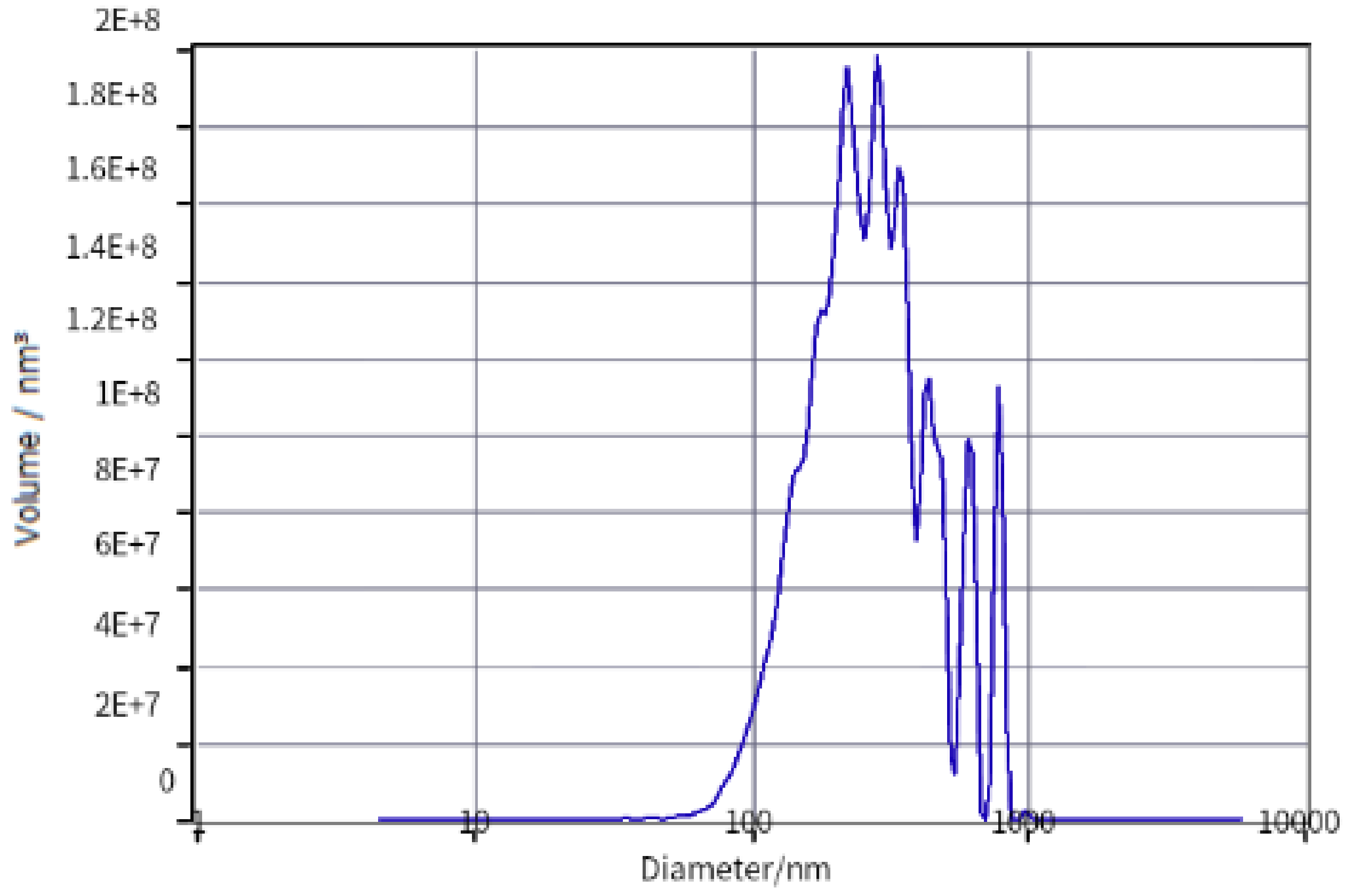

PeakAnalysis(Concentration)

Diameter/nm	Particles/mL	FWHM/nm	Percentage
139.0	2.6E+6	154.6	100.0

XValues(allsizesaregiveninnm)

	Number	Concentration	Volume
X10	79.2	79.2	141.4
X50	134.2	134.2	267.5
X90	243.1	243.1	566.7
Span	1.2	1.2	1.6
Mean	153.6	153.6	313.2
StdDev	76.1	76.1	168.0

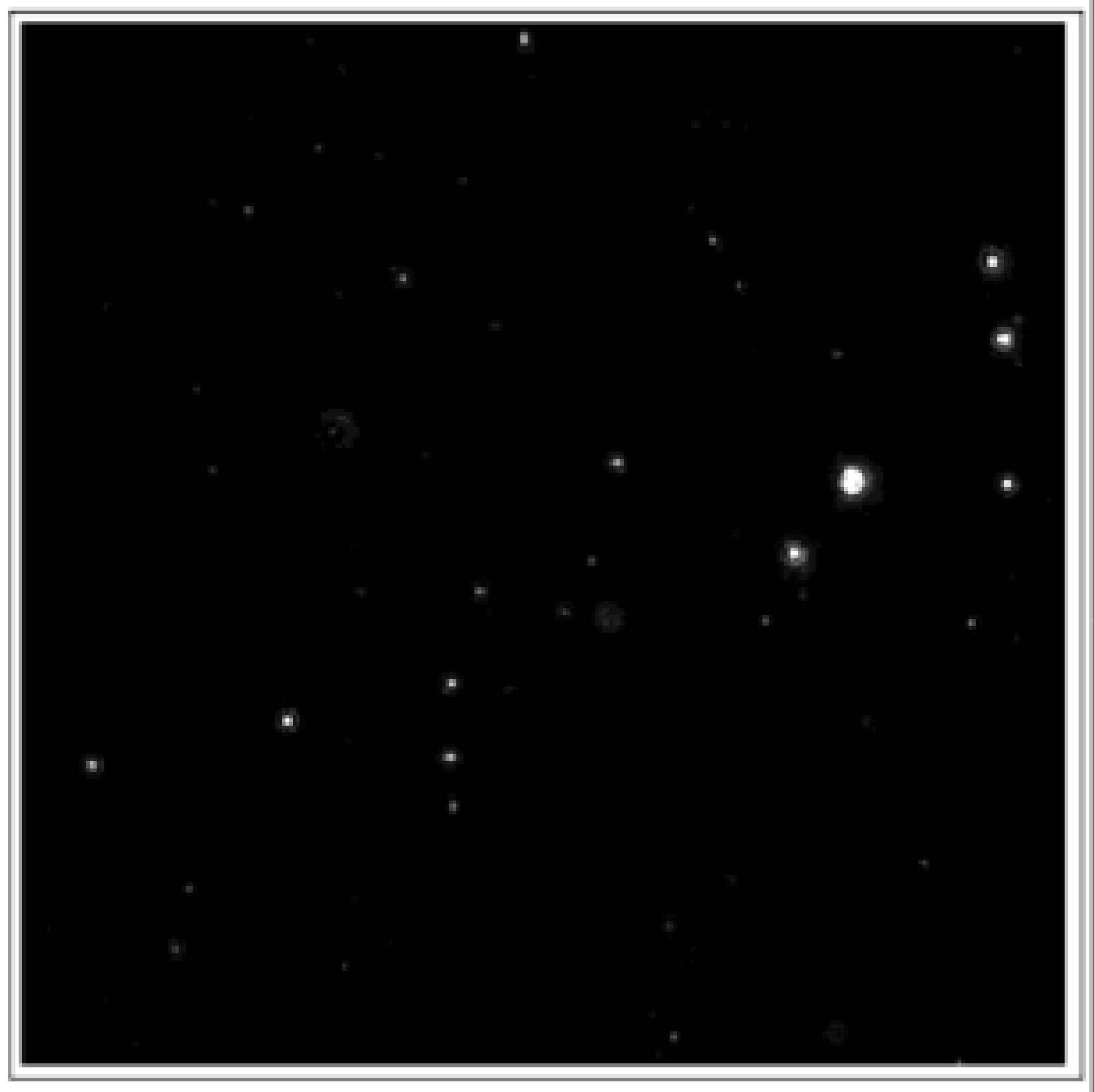

Comment

(Signature)
