# Supplementary material for: Diagnostic and differential diagnostic value of plasma extracellular vesicle-associated miRNAs in AIDS patients with tuberculosis and non-tuberculous mycobacterial infection
Source: Front Cell Infect Microbiol. 2026 Jul 28;16:1828046. doi: 10.3389/fcimb.2026.1828046 (PMC13456989; doi:10.3389/fcimb.2026.1828046)
Supplement: Supplementary file 3 [file SupplementaryFile3.pdf]

SampleParameters
SampleName:TB11
Comment:PS100nm/dilution1:250,000/520nmlaser
SampleRemarks0:
SampleRemarks1:
SampleRemarks2:
Electrolyte:PBS
Temperature:20.24°Csensed
pH7.0entered

InstrumentParameters
LaserWavelength:520nm
FilterWavelength:Scatter

SOP:Ev
SizeDistribution1Cycle11Positions

Result(sizesinnm)

	Number	Concentration	Volume
Median(X50)	135.0	135.0	262.9
Span	75.0	75.0	274.2

Concentration:	8.2E+7Particles/mL
DilutionFactor:	200
OriginalConcentration:	1.6E+10Particles/mL

Quality
AverageCountedParticlesperFrame:200
NumberofTracedParticles:1733

AnalysisParameters
MaxArea:1000,MinArea:5,MinBrightness:20

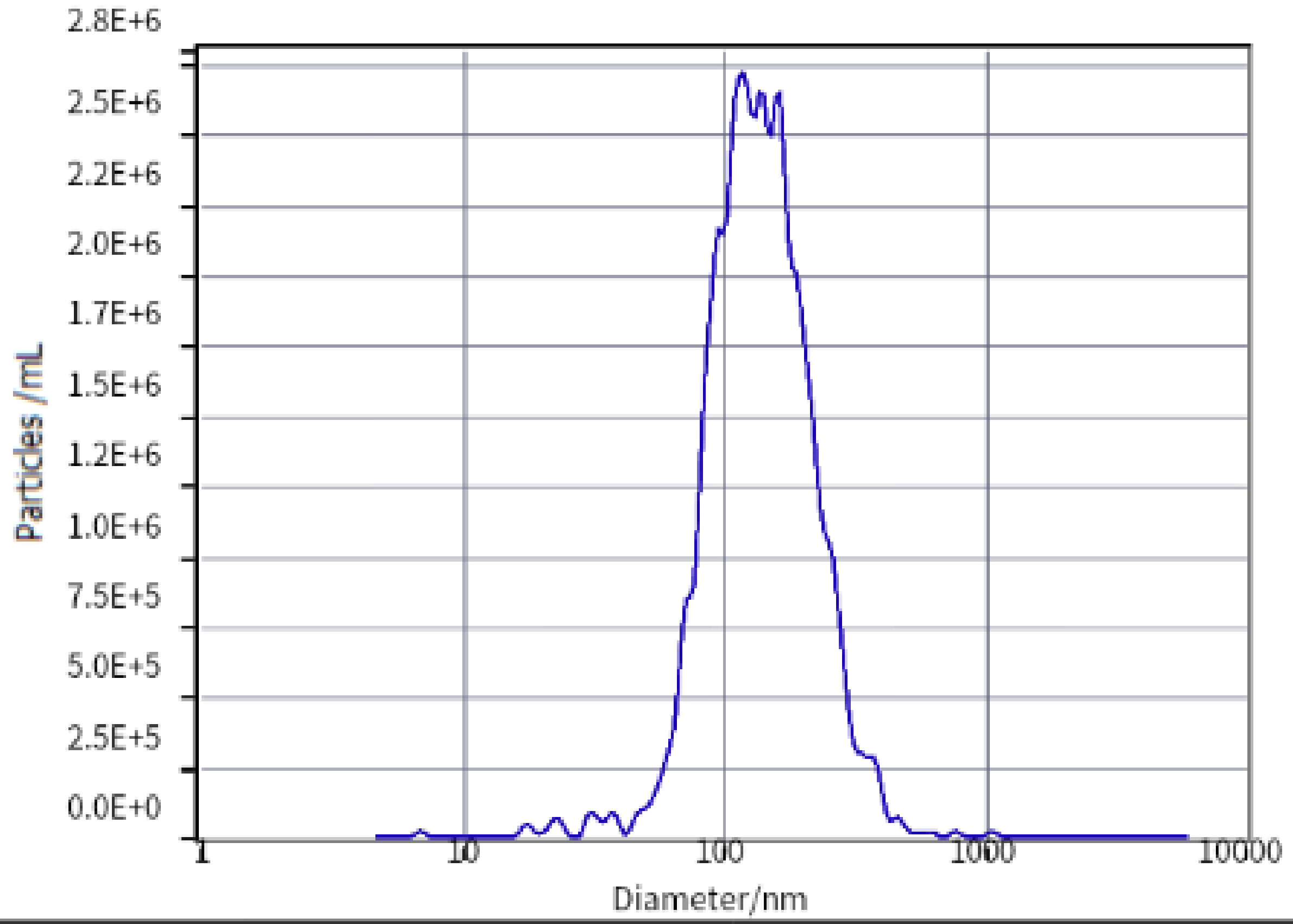

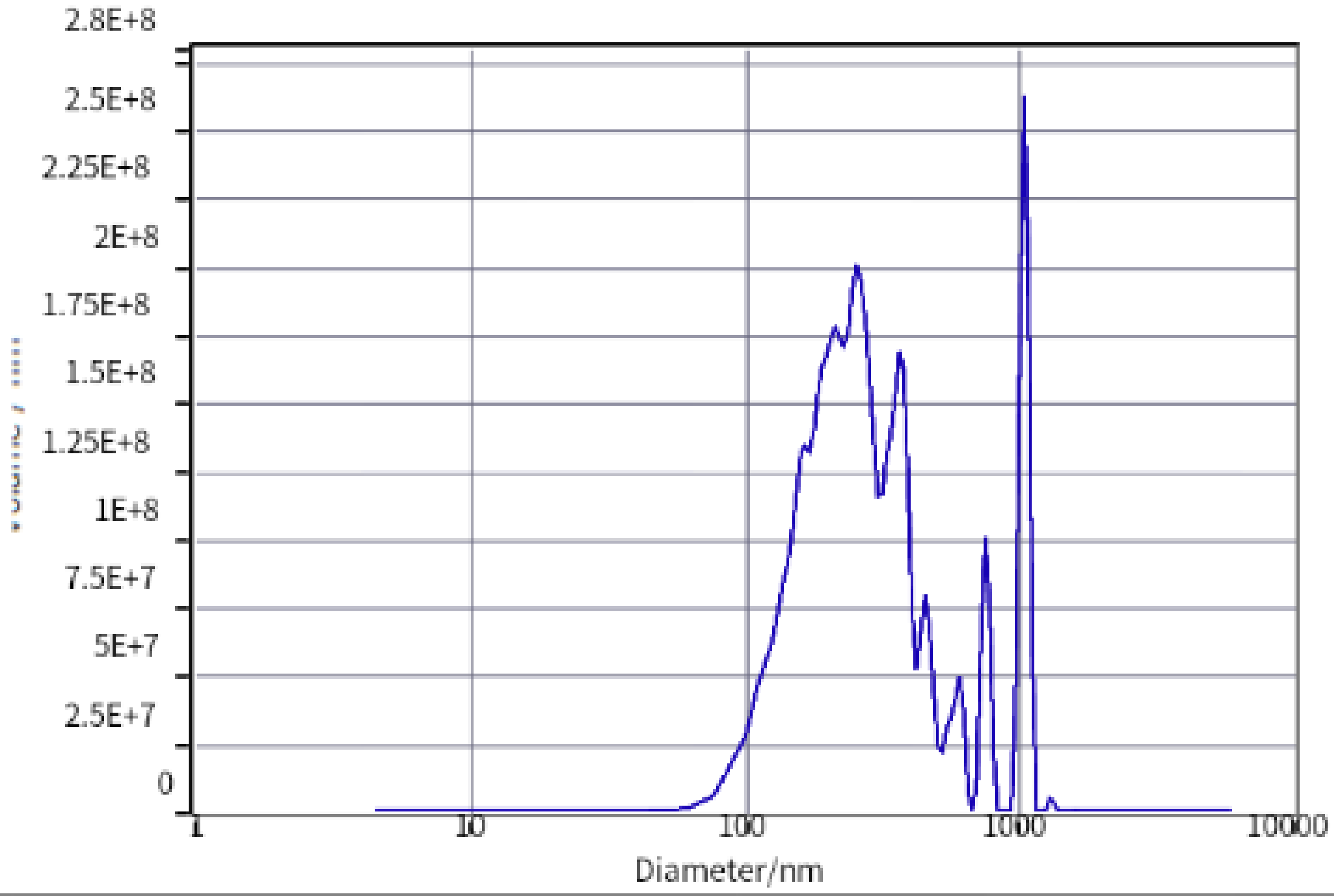

PeakAnalysis(Concentration)

Diameter/nm	Particles/mL	FWHM/nm	Percentage
158.8	2.6E+6	85.2	47.9
118.4	2.7E+6	41.4	42.6
137.2	2.6E+6	9.6	9.5

XValues(allsizesaregiveninnm)

	Number	Concentration	Volume
X10	80.6	80.6	141.6
X50	135.0	135.0	262.9
X90	238.7	238.7	773.9
Span	1.2	1.2	2.4
Mean	152.6	152.6	367.2
StdDev	75.0	75.0	274.2

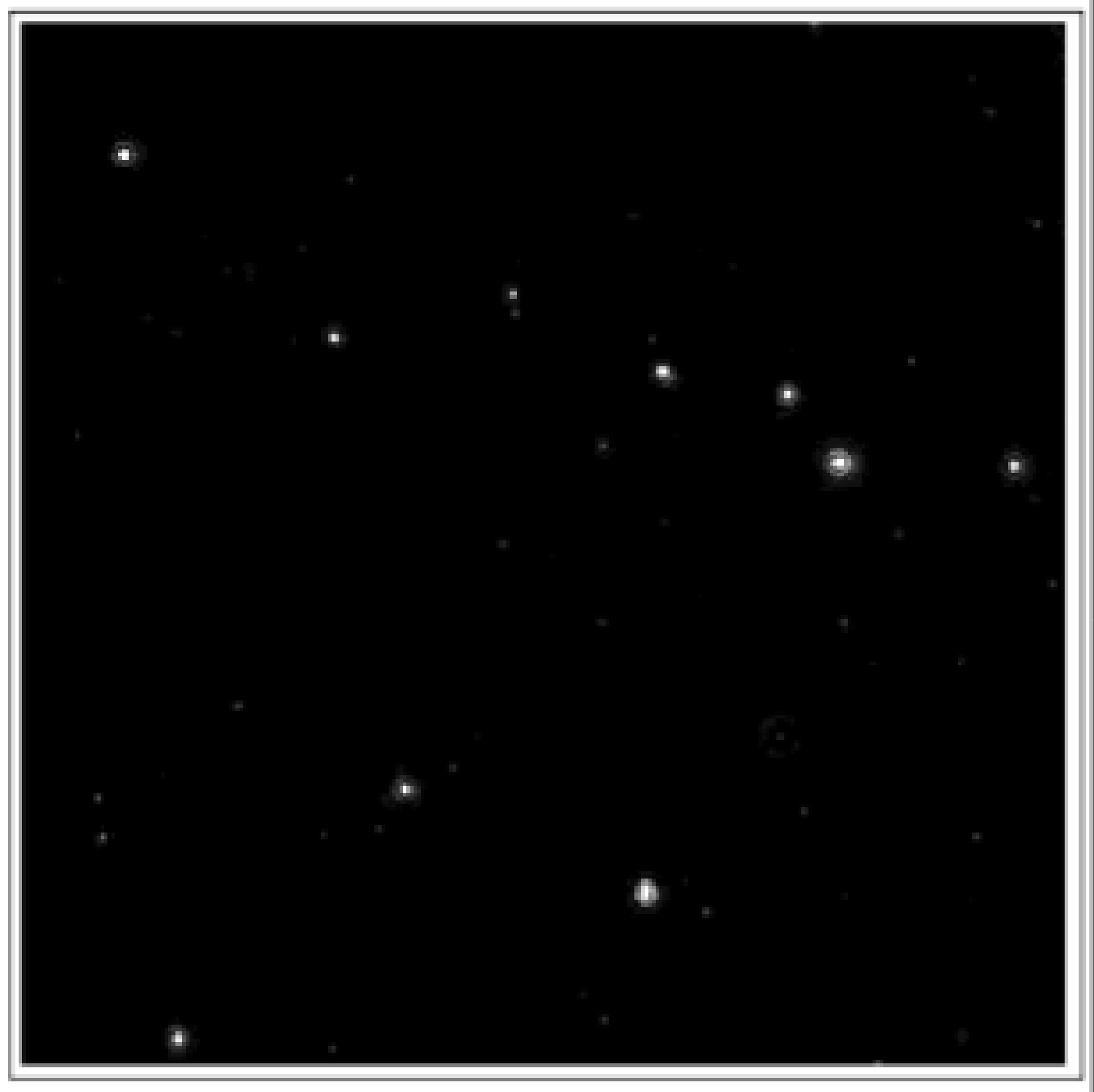

Comment

(Signature)
