# Supplementary material for: Diagnostic and differential diagnostic value of plasma extracellular vesicle-associated miRNAs in AIDS patients with tuberculosis and non-tuberculous mycobacterial infection
Source: Front Cell Infect Microbiol. 2026 Jul 28;16:1828046. doi: 10.3389/fcimb.2026.1828046 (PMC13456989; doi:10.3389/fcimb.2026.1828046)
Supplement: Supplementary file 4 [file Table4.docx]

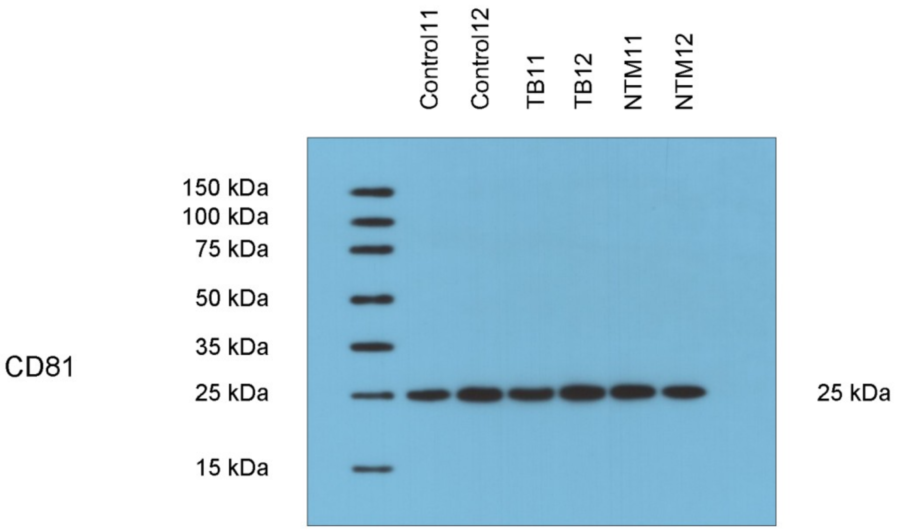
Control11
Control12
TB11
TB12
NTM11
NTM12
150 kDa
100 kDa
75 kDa
50 kDa
35 kDa
CD81
25 kDa
15 kDa
25 kDa

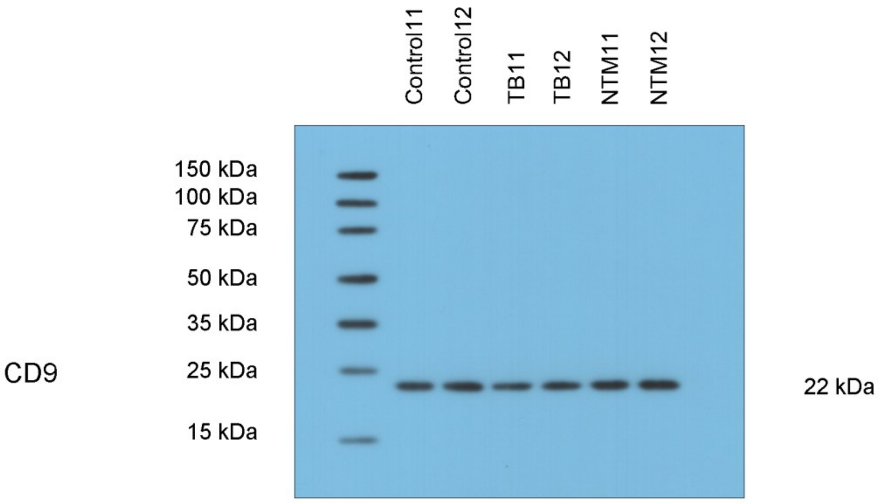
Control11
Control12
TB11
TB12
NTM11
NTM12
150 kDa
100 kDa
75 kDa
50 kDa
35 kDa
CD9
25 kDa
15 kDa
22 kDa

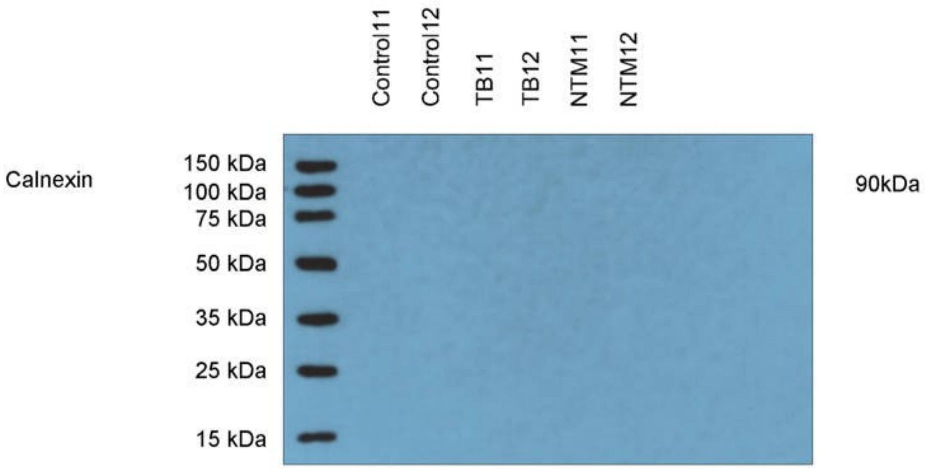
Control11
Control12
TB11
TB12
NTM11
NTM12
Calnexin
150 kDa
100 kDa
75 kDa
50 kDa
35 kDa
25 kDa
15 kDa
90kDa
