# Supplementary material for: Diagnostic and differential diagnostic value of plasma extracellular vesicle-associated miRNAs in AIDS patients with tuberculosis and non-tuberculous mycobacterial infection
Source: Front Cell Infect Microbiol. 2026 Jul 28;16:1828046. doi: 10.3389/fcimb.2026.1828046 (PMC13456989; doi:10.3389/fcimb.2026.1828046)
Supplement: Supplementary file 4 [file Table2.docx]

**Supplementary Table 1. Diagnostic characteristics of the validation cohort (n=110)**

| **Group** | **n** | **Confirmation method** | **n (%)** | **Sampling time** |
| --- | --- | --- | --- | --- |
| AIDS/TB | 30 |  |  | After admission, before treatment |
|  |  | Sequencing-confirmed  *M. tuberculosis* | 24 (80%) |  |
|  |  | Liquid culture + MPB64 positive+ clinical | 6 (20%) |  |
| AIDS/NTM | 50 |  |  | After admission, before treatment |
|  |  | Sequencing-confirmed  *M. avium* | 17 (34%) |  |
|  |  | Sequencing-confirmed  *M. intracellulare* | 3 (6%) |  |
|  |  | Sequencing-confirmed  *M. kansasii* | 6 (12%) |  |
|  |  | Liquid culture + MPB64 negative + clinical | 24 (48%) |  |
| AIDS Control | 30 | No mycobacterial infection | 30 (100%) | After admission |

Footnotes: All patients were treatment-naïve at the time of plasma collection. Blood samples were collected as soon as possible after hospital admission and prior to initiation of anti-mycobacterial therapy. Diagnosis of TB was based on liquid culture (MGIT), MPB64 antigen test, and/or sequencing, combined with clinical and radiological findings. MPB64 positivity distinguishes M. tuberculosis complex from NTM. For NTM patients, MPB64 negativity along with culture positivity was used for initial identification, with species confirmation by sequencing in 26 cases.
