# Supplementary material for: Diagnostic and differential diagnostic value of plasma extracellular vesicle-associated miRNAs in AIDS patients with tuberculosis and non-tuberculous mycobacterial infection
Source: Front Cell Infect Microbiol. 2026 Jul 28;16:1828046. doi: 10.3389/fcimb.2026.1828046 (PMC13456989; doi:10.3389/fcimb.2026.1828046)
Supplement: Supplementary file 5 [file Table3.docx]

**Supplementary Table 2. Individual 2<sup>−ΔΔCt</sup> values of plasma EV-associated miRNAs in the validation cohort (n=110)**

| Sample ID | Group | miR-374a-5p (2<sup>−ΔΔCt</sup>) | miR-652-3p (2<sup>−ΔΔCt</sup>) | let-7c-5p (2<sup>−ΔΔCt</sup>) |
| --- | --- | --- | --- | --- |
| 1-Control1 | Control | 0.29 | 1.35 | 1.08 |
| 1-Control2 | Control | 0.34 | 1.33 | 1.09 |
| 2-DZZ3 | Control | 0.74 | 1.09 | 0.59 |
| 2-DZZ4 | Control | 0.66 | 0.97 | 0.66 |
| 2-DZZ5 | Control | 0.46 | 0.99 | 1.53 |
| 2-DZZ6 | Control | 0.38 | 0.90 | 0.84 |
| 2-DZZ7 | Control | 1.17 | 0.96 | 0.77 |
| 2-DZZ8 | Control | 0.42 | 0.92 | 0.99 |
| 2-DZZ9 | Control | 0.73 | 0.95 | 2.46 |
| 2-DZZ10 | Control | 0.63 | 0.95 | 1.27 |
| 3-DZZ1 | Control | 1.00 | 1.00 | 1.00 |
| 3-DZZ2 | Control | 1.36 | 1.10 | 0.97 |
| 3-DZZ3 | Control | 1.15 | 0.99 | 0.95 |
| 3-DZZ4 | Control | 1.72 | 1.41 | 0.86 |
| 3-DZZ5 | Control | 1.24 | 1.18 | 1.25 |
| 3-DZZ6 | Control | 0.96 | 1.27 | 1.31 |
| 3-DZZ7 | Control | 1.18 | 1.47 | 1.05 |
| 3-DZZ8 | Control | 1.13 | 1.28 | 1.11 |
| 3-DZZ9 | Control | 1.32 | 1.18 | 0.89 |
| 3-DZZ10 | Control | 1.31 | 1.57 | 1.16 |
| 4-Control11 | Control | 36.44 | 0.99 | 0.35 |
| 4-Control12 | Control | 26.56 | 1.04 | 0.46 |
| 4-Control13 | Control | 32.62 | 1.09 | 0.40 |
| 4-Control14 | Control | 28.47 | 1.29 | 0.40 |
| 4-Control15 | Control | 37.76 | 1.13 | 0.41 |
| 4-Control16 | Control | 30.88 | 1.28 | 0.54 |
| 4-Control17 | Control | 30.53 | 1.27 | 0.39 |
| 4-Control18 | Control | 23.58 | 1.46 | 0.47 |
| 4-Control19 | Control | 32.09 | 1.22 | 0.52 |
| 4-Control20 | Control | 33.29 | 1.41 | 0.43 |
| 1-TB-1 | TB | 0.50 | 0.83 | 8.80 |
| 1-TB-2 | TB | 0.37 | 0.67 | 9.41 |
| 2-TB-3 | TB | 0.27 | 1.12 | 1.95 |
| 2-TB-4 | TB | 0.23 | 0.58 | 5.27 |
| 2-TB-5 | TB | 0.18 | 0.67 | 3.75 |
| 2-TB-6 | TB | 0.21 | 0.68 | 4.70 |
| 2-TB-7 | TB | 0.28 | 0.72 | 2.87 |
| 2-TB-8 | TB | 0.19 | 0.49 | 3.34 |
| 2-TB-9 | TB | 0.24 | 0.60 | 3.14 |
| 2-TB-10 | TB | 0.42 | 0.75 | 2.69 |
| 3-TB1 | TB | 0.27 | 0.75 | 2.66 |
| 3-TB2 | TB | 0.34 | 0.74 | 2.89 |
| 3-TB3 | TB | 0.36 | 0.62 | 2.87 |
| 3-TB4 | TB | 0.32 | 0.75 | 3.48 |
| 3-TB5 | TB | 0.29 | 0.84 | 3.15 |
| 3-TB6 | TB | 0.22 | 0.67 | 4.14 |
| 3-TB7 | TB | 0.33 | 0.70 | 2.66 |
| 3-TB8 | TB | 0.26 | 0.55 | 3.86 |
| 3-TB9 | TB | 0.30 | 0.63 | 2.15 |
| 3-TB10 | TB | 0.44 | 0.94 | 2.96 |
| 4-TB11 | TB | 35.79 | 1.41 | 0.32 |
| 4-TB12 | TB | 28.98 | 1.65 | 0.34 |
| 4-TB13 | TB | 35.60 | 1.47 | 0.31 |
| 4-TB14 | TB | 35.01 | 1.70 | 0.30 |
| 4-TB15 | TB | 33.16 | 1.41 | 0.32 |
| 4-TB16 | TB | 33.05 | 1.54 | 0.35 |
| 4-TB17 | TB | 34.98 | 1.71 | 0.36 |
| 4-TB18 | TB | 31.68 | 1.69 | 0.34 |
| 4-TB19 | TB | 32.80 | 1.58 | 0.32 |
| 4-TB20 | TB | 32.35 | 1.55 | 0.33 |
| 1-BN-1 | NTM | 0.21 | 0.46 | 9.29 |
| 1-BN-2 | NTM | 0.26 | 0.46 | 9.50 |
| 1-NFZ-1 | NTM | 0.38 | 0.46 | 10.97 |
| 1-NFZ-2 | NTM | 0.25 | 0.36 | 9.80 |
| 1-KSS-1 | NTM | 0.26 | 0.47 | 10.10 |
| 1-KSS-2 | NTM | 0.21 | 0.39 | 9.27 |
| 2-NTM7 | NTM | 0.06 | 0.41 | 25.58 |
| 2-NTM8 | NTM | 0.57 | 0.58 | 6.95 |
| 2-NTM9 | NTM | 0.38 | 0.45 | 5.63 |
| 2-NTM10 | NTM | 0.22 | 0.43 | 6.09 |
| 2-NTM11 | NTM | 0.16 | 0.23 | 5.14 |
| 2-NTM12 | NTM | 0.18 | 0.19 | 5.65 |
| 2-NTM13 | NTM | 0.88 | 0.58 | 6.19 |
| 2-NTM14 | NTM | 0.81 | 0.53 | 16.70 |
| 2-NTM15 | NTM | 0.16 | 0.34 | 12.49 |
| 2-NTM16 | NTM | 0.43 | 0.38 | 8.51 |
| 2-NTM17 | NTM | 0.47 | 0.24 | 6.34 |
| 2-NTM18 | NTM | 0.27 | 0.21 | 5.84 |
| 2-NTM19 | NTM | 0.26 | 0.55 | 5.53 |
| 2-NTM20 | NTM | 0.40 | 0.35 | 13.48 |
| 2-NTM21 | NTM | 0.51 | 0.31 | 10.08 |
| 2-NTM22 | NTM | 0.33 | 0.28 | 8.29 |
| 2-NTM23 | NTM | 0.29 | 0.23 | 6.11 |
| 2-NTM24 | NTM | 0.19 | 0.22 | 7.93 |
| 2-NTM25 | NTM | 0.21 | 0.52 | 6.84 |
| 2-NTM26 | NTM | 0.42 | 0.43 | 13.86 |
| 2-NTM27 | NTM | 0.06 | 0.31 | 8.99 |
| 2-NTM28 | NTM | 0.23 | 0.36 | 8.72 |
| 2-NTM29 | NTM | 0.13 | 0.31 | 8.28 |
| 2-NTM30 | NTM | 0.37 | 0.23 | 4.93 |
| 3-NTM1 | NTM | 0.24 | 0.52 | 8.70 |
| 3-NTM2 | NTM | 0.27 | 0.51 | 9.94 |
| 3-NTM3 | NTM | 0.24 | 0.53 | 9.88 |
| 3-NTM4 | NTM | 0.28 | 0.60 | 9.82 |
| 3-NTM5 | NTM | 0.27 | 0.57 | 9.40 |
| 3-NTM6 | NTM | 0.22 | 0.52 | 9.27 |
| 3-NTM7 | NTM | 0.22 | 0.48 | 10.99 |
| 3-NTM8 | NTM | 0.22 | 0.57 | 8.02 |
| 3-NTM9 | NTM | 0.24 | 0.56 | 10.37 |
| 3-NTM10 | NTM | 0.17 | 0.53 | 8.58 |
| 4-NTM11 | NTM | 2.19 | 0.47 | 0.59 |
| 4-NTM12 | NTM | 2.02 | 0.45 | 0.57 |
| 4-NTM13 | NTM | 1.66 | 0.51 | 0.56 |
| 4-NTM14 | NTM | 1.85 | 0.44 | 0.56 |
| 4-NTM15 | NTM | 2.00 | 0.46 | 0.54 |
| 4-NTM16 | NTM | 1.90 | 0.51 | 0.53 |
| 4-NTM17 | NTM | 2.00 | 0.42 | 0.53 |
| 4-NTM18 | NTM | 2.15 | 0.45 | 0.53 |
| 4-NTM19 | NTM | 1.81 | 0.51 | 0.48 |
| 4-NTM20 | NTM | 1.79 | 0.50 | 0.52 |

Abbreviations: TB, AIDS patients with tuberculosis; NTM, AIDS patients with non-tuberculous mycobacterial infection; Control, AIDS patients without mycobacterial infection; 2<sup>−ΔΔCt</sup> values were normalized to U6 snRNA and calibrated to the mean of the AIDS control group (with 3-DZZ1 as the reference calibrator). Raw Ct values are provided in Supplementary File 1.
